# Dual-targeted delivery of temozolomide by multi-responsive nanoplatform via tumor microenvironment modulation for overcoming drug resistance to treat glioblastoma

**DOI:** 10.1186/s12951-024-02531-3

**Published:** 2024-05-17

**Authors:** Xiaojie Chen, Yuyi Zheng, Qi Zhang, Qi Chen, Zhong Chen, Di Wu

**Affiliations:** Key Laboratory of Neuropharmacology and Translational Medicine of Zhejiang Province, The First Affiliated Hospital, School of Pharmaceutical Sciences, Zhejiang Chinese Medical University, Hangzhou, 310053 China

**Keywords:** Glioblastoma, Blood-brain barrier, Dual-targeted delivery, Temozolomide, Drug resistance, Tumor microenvironment

## Abstract

Glioblastoma (GBM) is the most aggressive primary brain tumor with low survival rate. Currently, temozolomide (TMZ) is the first-line drug for GBM treatment of which efficacy is unfortunately hindered by short circulation time and drug resistance associated to hypoxia and redox tumor microenvironment. Herein, a dual-targeted and multi-responsive nanoplatform is developed by loading TMZ in hollow manganese dioxide nanoparticles functionalized by polydopamine and targeting ligands RAP12 for photothermal and receptor-mediated dual-targeted delivery, respectively. After accumulated in GBM tumor site, the nanoplatform could respond to tumor microenvironment and simultaneously release manganese ion (Mn^2+^), oxygen (O_2_) and TMZ. The hypoxia alleviation via O_2_ production, the redox balance disruption via glutathione consumption and the reactive oxygen species generation, together would down-regulate the expression of O^6^-methylguanine-DNA methyltransferase under TMZ medication, which is considered as the key to drug resistance. These strategies could synergistically alleviate hypoxia microenvironment and overcome TMZ resistance, further enhancing the anti-tumor effect of chemotherapy/chemodynamic therapy against GBM. Additionally, the released Mn^2+^ could also be utilized as a magnetic resonance imaging contrast agent for monitoring treatment efficiency. Our study demonstrated that this nanoplatform provides an alternative approach to the challenges including low delivery efficiency and drug resistance of chemotherapeutics, which eventually appears to be a potential avenue in GBM treatment.

**Supplementary Information:**

The online version contains supplementary material available at 10.1186/s12951-024-02531-3.

## Introduction

As the grade IV form of glioma, glioblastoma (GBM) is the most aggressive primary brain tumor, which accounts for 45.2% of all malignant central nervous system tumors, and the median survival rate after diagnosis is only 16–18 months [1–5]. Current standard of care for GBM treatment is surgical resection, followed by radiotherapy with temozolomide (TMZ)-mediated chemotherapy [6, 7].

TMZ, an alkylating agent, is the front-line chemotherapeutic drug in GBM treatment which has been approved by FDA in 1995 [8]. However, the therapeutic outcome is limited by its short blood circulation time, lower tumor accumulation, and drug resistance [9, 10]. Increased expression of O^6^-methylguanine-DNA methyltransferase (MGMT), a DNA repair protein, is considered as the major mechanism of TMZ resistance. It is expected to restore the TMZ-induced DNA lesions, resulting in the compromised therapeutic effects [11–13]. In recent years, a number of strategies have been utilized to decrease MGMT expression and reverse TMZ resistance, such as administration of MGMT inhibitors (e.g., lomeguatrib) [14, 15], DNA repair inhibitors (e.g., O^6^-benzylguanine) [16], anti-cancer drugs (e.g., cisplatin and artesunate) [8, 17], and small interfering RNA [11, 18]. However, the anti-resistance effect will be strictly dependent on the loading efficiency of abovementioned therapeutics. As a common feature of tumor, hypoxia in GBM that plays a pivotal role in angiogenesis, invasiveness, and metastasis of tumor cells, is another hallmark in drug resistance against chemotherapy with MGMT overexpression [19–21]. Besides, the redox homeostasis between glutathione (GSH) and reactive oxygen species (ROS) is also related to drug resistance, which may have significant effects on drug metabolism of alkylating agents and activation of protective cellular mechanisms [22–24]. Hence, decreasing MGMT expression via alleviating hypoxia environment and disturbing redox homeostasis, is a promising strategy of overcoming drug resistance of TMZ.

As tumor microenvironment (TME)-responsive nanomaterials, nanoparticulated manganese dioxide (MnO_2_) has recently attracted extensive attention acting as a theranostic system for hypoxia alleviation and redox homeostasis regulation. Due to its sensitive activity with hydrogen peroxide (H_2_O_2_) and GSH, MnO_2_ can be catalyzed to produce oxygen (O_2_) and manganese ion (Mn^2+^) [25–29]. The released O_2_ can alleviate tumor hypoxia to decrease drug resistance of cancers. Meanwhile, the generated Mn^2+^ could not only serve as contrast agents for magnetic resonance imaging (MRI), but also catalyze endogenous H_2_O_2_ into hydroxyl radicals (·OH) with high cytotoxicity via chemodynamic therapy (CDT), making tumor cells more susceptible to chemotherapy. Compared with free Mn^2+^, MnO_2_ featured less Mn-based neurotoxicity because it could release Mn^2+^ only at the pathological area in an on-demand manner [30]. Therefore, MnO_2_ is a potential candidate of nanoplatform for brain molecular imaging and combined tumor therapy. Until now, MnO_2_-based nanomaterials have attracted extensive attention in solid tumors treatment, but rarely reported in glioma or GBM therapy [31–38], which is owing to the presence of blood-brain barrier (BBB) and blood-brain tumor barrier (BBTB). Hence, a BBB/BBTB permeable MnO_2_-based nanomaterial is anticipated to improve the GBM therapeutic efficacy.

BBB is a natural physical barrier in the brain composed of brain capillary endothelia cells and tight junctions, playing an essential role in protecting the brain from toxins and pathogens [39–41]. However, BBB not only positions in an uncontrolled range for surgical resection, but also poses a hamper for drug delivery [42, 43]. Importantly, BBB could transform into BBTB along with GBM tumor growth. BBTB is a structural-functional barrier located between microvasculature and tumor site, which further aggravates the inefficient drug delivery [44, 45]. Therefore, exploring an alternative strategy that can penetrate both BBB and BBTB, and target GBM cells is essential to improve GBM treatment. Recently, many efforts have been devoted to the development of BBB/BBTB crossing and GBM-targeted delivery, such as adsorptive-mediated transcytosis, carrier-mediated transcytosis, modulation of tight junctions, and etc [42, 46, 47]. Among all, near-infrared (NIR) irradiation, a non-invasive and reversible strategy, could cause local mild hyperthermia to safely realize transient opening of BBB, and enhance drug accumulation at the brain [48–50]. Besides, as an active transport strategy, receptor-mediated transcytosis (RMT) has been widely investigated in brain drug delivery via clathrin- or caveolin-mediated endocytosis. It works on the basis of receptor-ligand interactions, including low-density lipoprotein receptor-related protein (LRP) receptor, transferrin receptor, integrin receptor, and etc [51]. Compared with single targeting strategies, dual-targeting delivery systems could further improve targeting efficiency of GBM. Yan et al. proposed a two-order targeted nanoprobe combining Angiopep-2 peptide with cyclic [RGDyK] peptide for GBM treatment, which could combinationally target BBB and BBTB via LRP and integrin receptor [52]. Another magnet and transferrin co-modified nanoparticle was constructed by Liu et al. for promoting deep penetration and increasing anti-GBM activity, demonstrating the advantages of dual-targeting strategies [53]. Here, we proposed that a combination of NIR irradiation and LRP transcytosis could facilitate the BBB crossing and enhance GBM treatment efficiency.

**Fig. 1:**
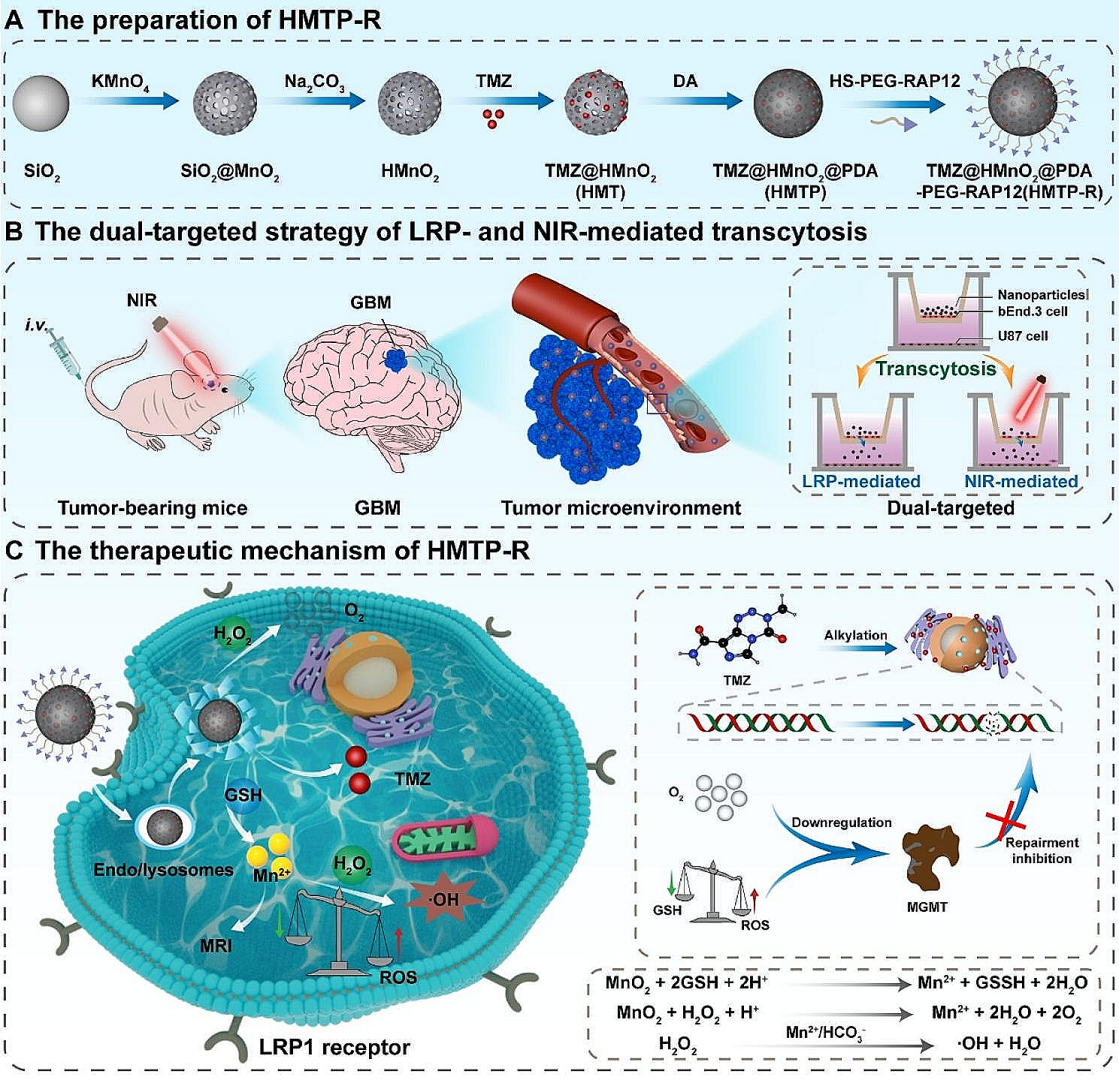
(**A**) Schematic illustration of the preparation of dual-targeted nanoparticles. (**B**) Scheme of the dual-targeted strategy of LRP- and NIR-mediated transcytosis. (**C**) Diagram of the therapeutic mechanism of HMTP-R against GBM

Herein, we fabricated a multi-responsive nanoplatform (TMZ@HMnO_2_@PDA-PEG-RAP12, HMTP-R) with dual-targeted delivery by synthesis of polydopamine (PDA)-coated hollow MnO_2_ nanoparticles (abbreviated as HMnO_2_@PDA, HMP) with targeting peptide receptor associated protein 12 (RAP12) to specifically recognize LRP1 receptor which is overexpressed on both BBB and tumor cells (Fig. 1A) [54–56]. Thanks to the satisfactory biocompatibility and photothermal conversion of PDA, the as-prepared nanoparticles could penetrate the BBB via a dual-targeted strategy of LRP- and NIR-mediated transcytosis (LRP-NIR) (Fig. 1B). Upon reaching the site, the TME-responsive MnO_2_ nanoparticles would respond to the pathological TME including mild acidy, high GSH level and ROS expression, and produce Mn^2+^ and O_2_. With simultaneous triggered TMZ releasing and GSH depleting [23, 57–59], the HMTP-R nanoplatform could alleviate hypoxia condition, down-regulate MGMT expression and promote tumor apoptosis, as well as monitor therapy via MRI (Fig. 1C). Collectively, this nanoplatform capable of “LRP-NIR” dual-targeted and multi-responsive properties provides a potential avenue for overcoming drug resistance and enhancing therapeutic efficiency of GBM treatment.

## Experimental section

### Materials

TMZ and L-GSH were purchased from Shanghai Macklin Biochemical Technology Co., Ltd. (Shanghai, China). Methylene blue (MB) and 2,7-dichlorofluorescein diacetate (DCFH-DA) were obtained from Beijing Solarbio Science & Technology Co., Ltd. (Beijing, China). Dopamine hydrochloride (DA‧HCl), 5,5-dithiobis (2-nitrobenzoic acid) (DTNB), and indocyanine green (ICG) were purchased from Aladdin (Shanghai, China). 4’,6-Diamidino-2-phenylindole (DAPI), Lyso-Tracker Red, and Hoechst 33,342 were obtained from Beyotime (Shanghai, China). D-luciferin potassium salt was purchased from BioFroxx (Germany). Calcein-AM/PI double staining kit was purchased from Yeasen Biotech Co., Ltd. (Shanghai, China). Annexin V-FITC/PI detection kit was purchased from Becton, Dickinson and Company (USA). The Luciferin (Luc) lentiviral vector was purchased from Shanghai Jikai Gene Chemical Technology Co., Ltd (GV260 vector, Shanghai, China). MGMT enzyme linked immunosorbent assay (Elisa) kit was obtained from Jiangsu Meimian Industrial Co., Ltd (Jiangsu, China).

### Preparation of TMZ@HMnO_2_@PDA (HMTP) nanoparticles

HMnO_2_ was synthesized following the previous method with some modifications [32]. Briefly, KMnO_4_ aqueous solution (5 mg/mL, 10 mL) was added dropwise to SiO_2_ solution (10 mg) under ultrasonication, and then stirred overnight. The obtained SiO_2_@MnO_2_ was collected by centrifugation, washed, and then reacted with Na_2_CO_3_ solution (2 M) at 60 ºC for 12 h to etch SiO_2_. HMnO_2_ was obtained after centrifugation, washed, and redispersed in water.

For TMZ loading, HMnO_2_ (6 mg) was mixed with various concentrations of TMZ aqueous solution (0.5, 1, 1.5 or 2 mg/mL, 6 mL) under vigorously stirring. After 24 h, the drug loading nanoparticles (TMZ@HMnO_2_, HMT) were collected after centrifugation, and washed with water. The supernatant was collected for drug loading (DL) and encapsulation efficiency (EE) evaluation.

For PDA coating, the HMT (6 mg) was redispersed in 6 mL of Tris-HCl buffer (10 mM, pH 8.5) containing DA‧HCl (6 mg), and stirred for 6 h at room temperature. The final product (TMZ@HMnO_2_@PDA, HMTP) was centrifuged (4000 rpm, 15 min) and washed for three times.

### Preparation of TMZ@HMnO_2_@PDA-PEG-RAP12 (HMTP-R)

For the synthesis of HS-PEG-RAP12, RAP12-NH_2_ peptide was conjugated to HS-PEG-NHS by NHS esterification. Briefly, HS-PEG-NHS (20 mg) and RAP12-NH_2_ (10 mg) were dissolved in NaHCO_3_-Na_2_CO_3_ buffer (2 mL), respectively. The mixture was allowed to react for 24 h at room temperature. Excessive reagent was removed by dialysis (MWCO 5000 Da) against water for another 48 h. After lyophilization, the pure HS-PEG-RAP12 was obtained.

HMTP-R was synthesized in an alkaline solution. HS-PEG-RAP12 (2 mg/mL, 12 mg) was added into HMTP (12 mg), and reacted for 24 h. The HMTP-R was obtained after centrifugation and washing. HS-PEG-NHS was utilized as non-targeted control group to decorate HMTP, and the obtained product was named as HMTP-PEG (HMTP-P).

### Characterization

Transmission electronic microscopy (TEM) and scanning electron microscopy (SEM) were used to observe the morphology, elemental mapping and element distribution of nanoparticles. The hydrodynamic diameter and zeta potential were measured on Malvern Zetasizer (Nano ZS-90, Malvern, UK). X-ray photoelectron spectroscopy (XPS) was utilized to determine the surface chemistry of HMnO_2_ and HMP. The UV-Vis absorbance was measured via a spectrophotometer (UH5700, HITACHI, Japan). Mass spectra was recorded by MALDI-TOF (UltrafeXtreme, Bruker, Germany). The amount of TMZ and Mn was investigated using a microplate reader (Multiskan Sky, Thermo, USA) at 328 nm and inductively coupled plasma (ICP), respectively, while the drug loading (DL) and encapsulation efficiency (EE) of TMZ were calculated using the following formulas.

$$\text{D}\text{L}\left(\text{\%}\right)=\frac{\text{W}\text{e}\text{i}\text{g}\text{h}\text{t} \,\text{o}\text{f} \,\text{l}\text{o}\text{a}\text{d}\text{e}\text{d} \,\text{T}\text{M}\text{Z}}{\text{W}\text{e}\text{i}\text{g}\text{h}\text{t} \,\text{o}\text{f} \,\text{t}\text{o}\text{t}\text{a}\text{l} \,\text{T}\text{M}\text{Z} \,\text{l}\text{o}\text{a}\text{d}\text{e}\text{d} \,\text{n}\text{a}\text{n}\text{o}\text{p}\text{a}\text{r}\text{t}\text{i}\text{c}\text{l}\text{e}\text{s}}\times 100\%$$

$$\text{E}\text{E}\left(\text{\%}\right)=\frac{\text{W}\text{e}\text{i}\text{g}\text{h}\text{t} \,\text{o}\text{f} \,\text{l}\text{o}\text{a}\text{d}\text{e}\text{d} \,\text{T}\text{M}\text{Z}}{\text{W}\text{e}\text{i}\text{g}\text{h}\text{t} \,\text{o}\text{f} \,\text{f}\text{e}\text{e}\text{d}\text{i}\text{n}\text{g} \,\text{T}\text{M}\text{Z}}\times 100\%$$

### Detection of ·OH generation

‧OH generation was verified via MB degradation experiment. Briefly, NaHCO_3_ (25 mM) solutions containing 10 µg/mL MB, 10 mM H_2_O_2_, and HMP (25–100 µg/mL) were incubated at 25, 37, 43 or 50 °C for 2 h. The absorbance change of MB at 665 nm was monitored via a microplate reader to evaluate the ‧OH generation.

In addition, the scavenging effect of GSH on ‧OH was performed in the presence or absence of GSH (concentration from 0 to 10 mM).

### Detection of GSH depletion

The Ellman’s reagent DTNB was utilized as an indicator to detect the -SH group in GSH. Briefly, HMP with various concentration (0–500 µg/mL) were added into GSH solution (2 mM). After incubation for 1, 2, 4, 8 h at room temperature, 0.1 mL supernatant was collected after centrifugation, followed by adding 50 µL DTNB solution (10 mM). After incubation for another 10 min, the absorbance change of GSH at 412 nm was recorded using a microplate reader.

### Detection of O_2_ generation

O_2_ generation was detected by the dissolved oxygen meter. HMP (100 µg/mL) was dispersed into PBS with various pH value containing 10 mM H_2_O_2_. A dissolved oxygen meter was used to monitor the O_2_ generation.

### In vitro photothermal effect

An infrared thermal imaging camera was used to record the temperature changes of HMP of various concentrations or power intensities when exposed to an 808-nm laser irradiation for 10 min (1 W/cm^2^). And the photothermal stability of HMP was conducted by irradiating with 808-nm laser for 10 min, followed by a natural cooling for another 15 min. This “on-off” cycle was repeated five times.

### In vitro drug release

The cumulative release of TMZ and Mn from HMTP was conducted by centrifugation assay as bellow: HMTP was dispersed in 1 mL of PBS with various pH values with or without GSH (10 mM) and H_2_O_2_ (1 mM), and incubated at 37 ºC with constant stirring. At predetermined time intervals, 0.1 mL supernatant was collected after centrifugation (10,000 rpm, 5 min) for analysis by a microplate reader at 328 nm and ICP, and replaced with fresh buffer to maintain the constant volume.

### Cell culture

The human glioblastoma U87 cells and mouse brain microvascular endothelial cells (bEnd.3) were purchased from ATCC. The human TMZ resistant U251-TR cells were obtained from Nanjing Caobenyuan Biology Science and Technology Co., Ltd (Nanjing, China), and cultured with DMEM medium containing TMZ (16 µg/mL) to maintain drug resistance. The stable U87-Luc cells were derived from the coculture of Luc lentiviral vector and U87 cells at 37 °C for 48 h, followed by screened in DMEM medium containing 2 µg/mL puromycin. All cells were cultured in an incubator of 5% CO_2_ at 37 ºC and supplemented with DMEM medium containing 10% fetal bovine serum and 1% penicillin and streptomycin.

### Cellular uptake and endo/lysosomal observation

U87 and bEnd.3 cells were seeded into 24-well plates at a density of 5 × 10^4^ cells per dish and incubated overnight. ICG labeled HMP-PEG (HMP-P) or HMP-PEG-RAP12 (HMP-R) (ICG concentration: 20 µg/mL) were incubated with cells over 2–4 h. After washed with PBS, the cells were fixed with 4% paraformaldehyde (PFA), followed by staining with DAPI for 15 min, and then visualized by confocal laser scanning microscope (CLSM, FV3000, Olympus, Japan). And the quantitative analysis of cellular uptake was analyzed by flow cytometry (CytoFLEX S, Beckman, USA). Besides, a part of cells was collected for TEM cell sample preparation. After that, the intracellular localization was observed by TEM.

In the competitive binding experiment, U87 and bEnd.3 cells were pre-treated with 200 µg/mL free RAP12-NH_2_ for 0.5 h. After withdrawing the medium, ICG labeled HMP-R was added and incubated for 2–4 h. The following operation was as same as the non-pretreatment groups.

For endo/lysosomal escape study, U87 cells were seeded in confocal dishes and incubated overnight. Then Cy5.5 labeled HMP-P or HMP-R nanoparticles were incubated with cells over 1, 4, 8–24 h. Afterwards, cells were washed with PBS and stained with Lyso-Tracker Red and Hoechst 33,342, respectively. The colocalization of nanoparticles with lysosomes was observed via CLSM.

### Intracellular ROS generation

DCFH-DA was used to determine the intracellular ROS generation. Briefly, U87 cells were seeded into 24-well plates at a density of 1 × 10^5^ cells and cultured overnight. After incubation with Mn-derived nanoparticles (Mn concentration: 20 µg/mL) or TMZ (200 µg/mL) for 6 h, U87 cells were washed with PBS and stained with DCFH-DA. Afterward, the intracellular ROS was observed by an inverted fluorescence microscope (Leica, DFC7000T, Germany).

### Intracellular O_2_ evaluation

The intracellular O_2_ was measured by an O_2_ probe [Ru(dpp)_3_]Cl_2_ (RDPP). U87 cells were seeded in 6-well plates at a density of 2 × 10^5^ cells per well. After cultured overnight, the cells were incubated with RDPP (10 µg/mL) for another 4 h. Then the cells were washed with PBS three time, followed by treated with PBS, TMZ, HMTP-P, HMTP-R or HMP (TMZ: 50 µg/mL; HMP: 25, 50, 100 µg/mL) under normal or hypoxic condition for 24 h. The intracellular O_2_ was evaluated based on the fluorescence of RDPP by an inverted fluorescence microscope.

### Intracellular GSH evaluation

U87 cells were seeded in 6-well plates at a density of 2 × 10^5^ cells and incubated overnight before use. Then the culture medium was placed with PBS, TMZ (50 µg/mL), HMTP-P, HMTP-R or HMP (25, 50, 100 µg/mL) for 24 h. After removing the medium and washed with PBS, 100 µL Triton X-100 lysis buffer was added to lyse the cells for 20 min. 50 µL supernatant was collected after centrifugation, and mixed with DTNB solution (0.4 mM) for 30 min. The intracellular GSH content was measured by a microplate reader at 412 nm.

### In vitro BBB model construction and BBB crossing efficiency evaluation

In vitro BBB model was constructed with bEnd.3 cells using a transwell plate. Briefly, bEnd.3 cells were implanted on the upper chamber of the transwell plate at the density of 1 × 10^4^ cells, and cultured for several days. The trans-endothelial electrical resistance (TEER) value was monitored by Millicell ERS (Millipore, USA) every day. When the TEER value was above 200 Ω‧cm^2^, U87 cells were seeded in the lower chamber, and cultured overnight. Then the Cy5.5-labeled HMTP-P or HMTP-R (Cy5.5 concentration: 20 µg/mL) were added in the upper chamber, and incubated for 24 h. Finally, U87 cells in the lower chamber were collected to study the BBB crossing efficiency via flow cytometry.

### 3D tumor spheroids

U87 cells were seeded into 96-well ultralow attachment plates at the density of 1 × 10^4^ cells. After 5 days, the 3D tumor spheroids were treated with ICG labeled HMP-P or HMP-R for 8 h. Then the 3D tumor spheroids were washed with PBS, followed by fixed with 4% PFA. The deep permeability of nanoparticles was investigated by CLSM.

### Live/dead staining assay

U87 cells were seeded in confocal microscopy dishes at the density of 5 × 10^5^ cells, and incubated overnight. Then the medium was replaced with fresh medium containing TMZ and HMTP (TMZ concentration: 50 µg/mL), respectively, and incubated under normal or hypoxic condition for 24 h. The cells were washed with PBS, followed by co-stained with Calcein-AM and PI at dark. Finally, the cells were investigated via CLSM.

### Cell apoptosis assay

To assess the cell apoptosis of the nanoparticles to U87 cells, cells were seeded in 6-well plates at the density of 5 × 10^5^ cells/well and cultured overnight. The cells were incubated with TMZ, HMTP-P, or HMTP-R (TMZ concentration: 50 µg/mL) for 24 h. Then the cells were harvested and washed with PBS, followed by co-stained with Annexin V-FITC and PI according to its protocol, and finally analyzed by flow cytometry.

### In vitro cytotoxicity assay

The cytotoxicity of nanoparticles to U87 cells were determined by CCK-8 assay. Briefly, U87 cells were seeded into 96-well plates at the density of 1 × 10^4^ cells per well, and incubated overnight. Then the medium was replaced with HMP or HMTP with various concentrations, respectively. After incubation for 24 h, the cell viability of U87 cells after treatment was detected by CCK-8 assay.

### MGMT protein expression evaluation

To investigate the drug resistance-related protein MGMT expression, cell immunofluorescence staining and Elisa were conducted on U87 and U251-TR cells. Briefly, U87 and U251-TR cells (1 × 10^5^ per well) were seeded into 24-well plates. After incubation for 24 h, the medium was replaced with fresh medium containing TMZ, HMP, HMTP-P or HMTP-R (TMZ concentration: 20 µg/mL) for 72 h. After fixed with PFA and then blocked with 3% bovine serum albumin for 2 h, the cells were subjected to immunofluorescence staining of MGMT monoclonal primary antibody at 4 ºC overnight. Then, the cells were washed with PBS and incubated with fluorescent dye-linked secondary antibody for 2 h, flowed by staining with DAPI for 10 min. Finally, the cells were imaged using CLSM. On the other hand, the cells were washed and collected after incubated with various formulations for 72 h. After lysed with RIPA lysis buffer, the MGMT protein expression was verified via Elisa.

### Establishment of U87 tumor-bearing nude mice model

Male Balb/c nude mice and male Balb/c mice (6–8 weeks, 20 ± 2 g) were purchased from Zhejiang Chinese Medical University Laboratory Animal Research Center. All animal experiments are implemented in accordance with the guidance of Zhejiang Chinese Medical University Animal Care and Use Committee. 2 × 10^5^ U87-Luc cells were carefully established by intracranial injection to Balb/c nude mice (right striatum, A/*P* = 0.8 mm, M/L = 1.6 mm, D/V = 3.0 mm). Establishment of the intracranial GBM model was affirmed via In vivo Imaging System (IVIS, PerkinElmer, USA) at seven days after inoculation of U87-Luc cells.

### In vivo fluorescence imaging, tissue biodistribution and pharmacokinetics

After seven days, Cy5.5 labeled HMP-P and HMP-R (Cy5.5 concentration: 2.5 mg/kg) were intravenously injected into GBM-bearing nude mice. And the fluorescence images were acquired predetermined time by IVIS. The nude mice in HMP-R group were treated with 808-nm laser irradiation (1 W/cm^2^, 10 min) 1 h post-injection. After 24 h post-injection, the major organs and brain were harvested for ex vivo fluorescence imaging. Brain tissues were embedded by optimal cutting temperature (OCT) compound, and sectioned into 20 μm slices. Finally, the brain slices were covered with DAPI for nucleus staining, and observed under fluorescence microscope (Leica, DFC7000T, Germany).

Balb/c mice were used for pharmacokinetic analysis. After injection of Cy5.5 labeled HMP-P and HMP-R, 10–50 µL of blood was taken from the retro-orbital plexus at selected time. The Cy5.5 signal intensity was determined using IVIS system, and the pharmacokinetic parameters were analyzed via DAS 2.0.

### In vitro/vivo MR imaging

The HMP nanoparticles solutions at different concentrations (Mn concentration: 0, 0.05, 0.1, and 0.2 mM) with various PBS values (pH 7.4 or 5.0), were mixed with 1 mM H_2_O_2_ and 10 mM GSH for 24 h. After centrifugation, the supernatants were scanned on a 9.0 T MRI scanner (Time Medical, 9 T/110) to measure the T1 magnetic relaxation time.

GBM-bearing nude mice were intravenously injected of HMP-R (Mn concentration: 2 mg/kg). At pre-determined time (0, 30, 60, 120, and 180 min), the mice were undergoing brain MRI.

### In vivo anti-tumor evaluation

U87-Luc tumor-bearing Balb/c nude mice were randomly divided into six groups (6 mice per group), and treated with saline, TMZ, HMTP-P or HMTP-R (TMZ concentration: 2.5 mg/kg) via tail vein injection every three days for four doses. The HMTP-P + laser and HMTP-R + laser groups were treated with 808-nm laser irradiation (1 W/cm^2^, 10 min) after 1 h post-injection. The body weight was monitored every three days, and IVIS imaging system was utilized to measure the tumor volumes via bioluminescence imaging. At day 22, the nude mice were sacrificed, and their major organs were collected and fixed with 4% PFA. After embedding in paraffin and sectioning, the major tissues and brains were stained with hematoxylin and eosin (H&E), TUNEL and MGMT for histological analysis.

### In vivo biosafety evaluation

The in vivo biosafety of nanoparticles was evaluated by hemolytic test, open field test and blood biochemistry.

Firstly, red blood cells (RBCs) were isolated from serum, centrifuged and washed with PBS for five times. After dilution to 2% with PBS, the RBCs suspension was added into equal volume of the HMP suspension in PBS at the concentrations ranging from 10 to 500 µg/mL, and incubated at 37 ºC for 1 h. Then supernatant was collected by centrifugation and recorded by a microplate reader at 414 nm. PBS and Triton X-100 treated solution was used as the negative and positive control, respectively. The hemolysis rate was calculated as following:

$$\text{H}\text{e}\text{m}\text{o}\text{l}\text{y}\text{s}\text{i}\text{s} \,\text{r}\text{a}\text{t}\text{e} \left(\text{\%}\right)=\frac{{\text{A}}_{\text{s}\text{a}\text{m}\text{p}\text{l}\text{e}}-{\text{A}}_{\text{P}\text{B}\text{S}}}{{\text{A}}_{\text{T}\text{r}\text{i}\text{t}\text{o}\text{n} \text{X}}-{\text{A}}_{\text{P}\text{B}\text{S}}}\times 100\text{\%}$$

Then, healthy Balb/c mice were intravenously injected with saline, TMZ, HMTP-P, or HMTP-R (TMZ concentration: 2.5 mg/kg) every three day for four doses in compliance with the treatment schedule. At day 22, the motion trail of mice was captured using open field test to further evaluate their spontaneous activities, including exploratory behavior, general locomotor activity, and anxiety [60].

After open field test, blood samples were collected through the eye socket of mice for hematological parameters analysis of liver function indicators including alanine aminotransferase (ALT), aspartate aminotransferase (AST), albumin (ALB) and total protein (TP), as well as kidney function indicators involving blood urea nitrogen (BUN) and uric acid (UA).

### Statistical analysis

The data were presented as mean ± standard deviation (SD). The statistical significance between groups were performed using SPSS Statistic 22 software (IBM, Armonk, NY). And the significance level between the groups were analyzed through one-way ANOVA Tukey’s multiple comparisons test, and marked as * for *P* < 0.05, ** for *P* < 0.01, and *** for *P* < 0.001.

## Results and discussion

### Preparation and characterization of HMTP-R nanoparticles

HMTP-R nanoparticles were obtained as illustrated in Fig. 1. SiO_2_ nanoparticles were utilized as a hard template, and a uniform layer of MnO_2_ was grown on their surface by reduction of KMnO_4_ to form MnO_2_-coated SiO_2_ core-shell nanoparticles (SiO_2_@MnO_2_) [32]. HMnO_2_ nanoshells were obtained after incubated with Na_2_CO_3_ solution at 60 ºC to remove SiO_2_ template. The anti-GBM drug (TMZ) was at the meantime encapsulated in the hollow chamber of HMnO_2_ nanoshells to prepare HMT. Afterward, HMT was decorated with PDA by self-polymerization of dopamine to form PDA-coated HMT nanoparticles (HMTP). Under mild alkaline condition, HMTP was decorated with brain-targeting peptide HS-PEG-RAP12 or non-targeting peptide HS-PEG-NHS through Schiff’s base reaction [61], yielding HMTP-R or HMTP-P nanoparticles.

**Fig. 2:**
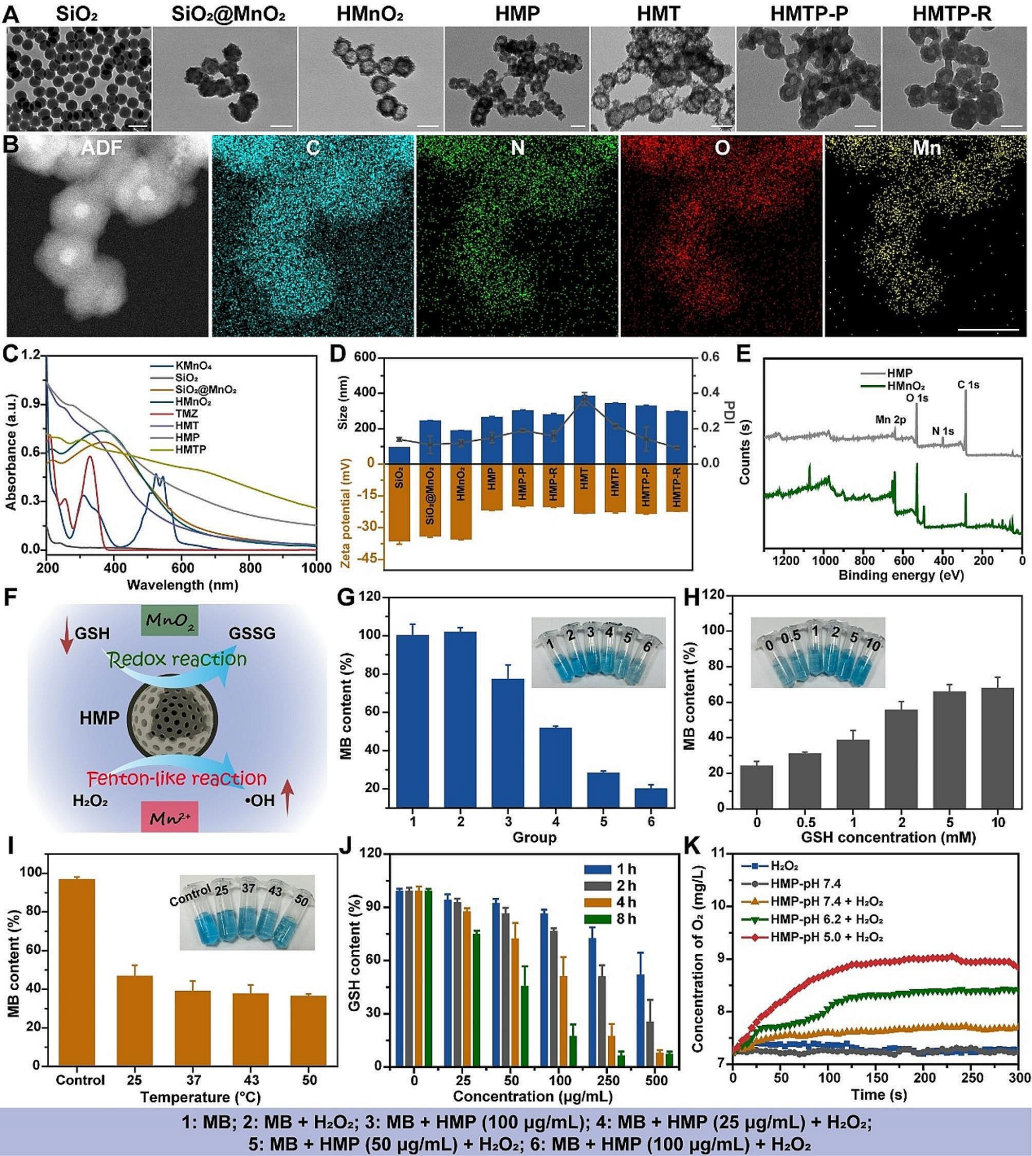
Characterization of HMP nanoparticles. (**A**) TEM images of SiO_2_, SiO_2_@MnO_2_, HMnO_2_, HMP, HMT, HMTP-P and HMTP-R. Scale bar: 100 nm. (**B**) Elemental mapping of HMP. Scale bar: 100 nm. (**C**) UV-Vis absorbance spectra of KMnO_4_, SiO_2_, SiO_2_@MnO_2_, HMnO_2_, TMZ, HMT, HMP and HMTP. (**D**) Hydrodynamic diameter, PDI and zeta potential of SiO_2_, SiO_2_@MnO_2_, HMnO_2_, HMP, HMP-P, HMP-R, HMT, HMTP, HMTP-P and HMTP-R (*n* = 3). (**E**) XPS survey spectrum of HMnO_2_ and HMP nanoparticles. (**F**) Schematic illustration for HMP-mediated redox and Fenton-like reaction. (**G**) ·OH generation recorded with MB as a probe by various groups in the presence of H_2_O_2_. (**H**) ·OH generation of HMP in the presence of H_2_O_2_ and different GSH concentrations (0–10 mM). (**I**) MB degradation by HMP in the presence of H_2_O_2_ after treated at different temperatures. Inset images from (**G**) to (**I**): corresponding photos reveal the ‧OH generation ability. (**J**) Time-dependent GSH consumption of HMP with various concentrations after 2 mM GSH treatment. (**K**) O_2_ generation from HMP added with H_2_O_2_ (10 mM) under different pH values. The data are presented as mean ± SD (*n* = 3)

Firstly, morphology of SiO_2_, SiO_2_@MnO_2_, HMnO_2_, and HMP was characterized by TEM as shown in Fig. 2A. We investigated the appropriate mass ratio of SiO_2_ and KMnO_4_. As displayed in Fig. S1, with the decrease of KMnO_4_, SiO_2_ nanoparticles could not be completely coated by the MnO_2_ layer. After etching, HMnO_2_ showed an obvious hollow structure at a ratio of 0.2:1 (Fig. 2A), which could further improve the drug loading of nanoparticles, while HMnO_2_ of other mass ratios exhibited collapsed nanostructure after etching. Hence, a mass ratio of 0.2:1 for SiO_2_:KMnO_4_ was used for further study. We also investigated the appropriate mass ratio of DA and HMnO_2_ via characterizations of TEM, hydrodynamic diameter, polydispersity index (PDI), zeta potential, size distribution, and photothermal ability. As shown in Fig. S2A-C, with the increasing of mass ratio, HMP nanoparticles were aggregated, and the hollow structure of HMnO_2_ has been completely covered by PDA. Besides, the photothermal ability of HMP was enhanced with the increase of mass ratio, which could mainly be attributed to the PDA coating (Fig. S2D). HMP owned similar temperature change curves after laser irradiation at the mass ratio of 0.5:1 and 1:1. Hence, HMP at the mass ratio of 1:1 was selected for further study, which showed uniform distribution, smaller size, hollow structure, and excellent photothermal ability. After self-polymerization of DA, a distinct two-layer structure could be observed in the TEM images of HMP, demonstrating successful coating of a PDA shell on HMnO_2_. Elemental mapping and energy dispersive X-ray spectroscopy (EDS) exhibited a uniform distribution of C, N, O, and Mn elements in HMP (Fig. 2B and Fig. S3). The mass analysis results from matrix-assisted laser desorption/ionization time-of-flight (MALDI-TOF) demonstrated the successful synthesis of HS-PEG-RAP12 (Fig. S4).

Next, ultraviolet-visible (UV-Vis) absorbance of the monomers and nanoparticles was evaluated via a UV-Vis spectrometer (Fig. 2C). After MnO_2_ coating and Na_2_CO_3_ etching, the absorbance spectra of SiO_2_@MnO_2_ and HMnO_2_ appeared a similar strong absorption at around 365 nm, which proved MnO_2_ coating. The addition of TMZ and PDA increased the absorbance of HMnO_2_ at UV and Vis absorption regions, respectively, confirming the TMZ encapsulation and PDA coating in/on HMnO_2_. Hydrodynamic diameter analysis showed a slight increase after HS-PEG-RAP12 peptide decoration from 263.23 ± 6.46 nm to 278.10 ± 7.79 nm, which were relatively larger than TEM images due to the presence of thicker hydration layer (Fig. 2D). The hydrodynamic diameter was increased from 187.23 ± 2.74 nm of HMnO_2_ to 382.93 ± 4.04 nm of HMT, and became aggregated with the PDI of 0.37 ± 0.04. The hydrodynamic diameter and PDI was decreased after self-polymerization of DA and PEG-ligand modification, which might be due to the enhanced dispersity of PDA and PEG coating. Furthermore, we investigated the dispersion stability of HMTP-P and HMTP-R in various medium including water, PBS, and DMEM containing 10% FBS. As shown in Fig. S5, there are no obvious change of HMTP-P and HMTP-R over 5 days in various medium, suggesting the good dispersion stability of HMTP-P and HMTP-R. Zeta potential analysis displayed an obvious increase from -35.20 ± 0.51 mV of HMnO_2_ to -19.97 ± 0.40 mV of HMP-R, which may enhance the cellular uptake of nanoparticles and reduce the non-specific protein adsorption. The decreased PDI further suggested their good dispersibility in water. XPS spectroscopy was utilized to demonstrate the formation of HMnO_2_ and HMP (Fig. 2E). Specifically, an N peak appeared in the XPS survey spectrum of HMP after PDA coating, and two remarkable peaks at 659.98 eV and 641.58 eV could be ascribed to Mn 2p^3/2^ and Mn 2p^1/2^ spin-orbit peaks of HMnO_2_, respectively (Fig. S6) [29].

HMP-mediated redox and Fenton-like reactions were illustrated in Fig. 2F. MB was selected as an indicator of ·OH generation which could be degraded by ·OH with a visible color change. As shown in Fig. 2G and Fig. S7A, MB absorbance was decreased with the increase of HMP concentration when incubated with H_2_O_2_ for 2 h. Remarkably, the CDT efficiency would be hampered by the existence of GSH, owning to the scavenging effects of GSH on ·OH [62]. The results showed that the MB absorbance was increased in the presence of GSH, and exhibited a concentration-dependent increase behavior (Fig. 2H and Fig. S7B), indicating that the ·OH generation would be influenced by GSH concentration, and the depletion of GSH could enhance CDT efficacy. Furthermore, the ·OH-induced MB degradation was facilitated at 50 °C compared with that at room temperature (Fig. 2I) [63]. MnO_2_ was also presenting GSH-consumption characteristic based on the DTNB-GSH standard curve (Figs. 2J, S8A and S9), further enhancing the ·OH-mediated CDT through disturbing the balance of tumor-related redox homeostasis regulation [64, 65]. In addition, MnO_2_ can produce O_2_ through catalyzing H_2_O_2_ in the acid environment, effectively alleviating the hypoxia of TME [30]. The O_2_ generation was monitored via a dissolved oxygen meter under different pH values with 10 mM H_2_O_2_. As shown in Fig. 2K, up to 9 mg/L of O_2_ was rapidly released by the reaction between HMP and H_2_O_2_ at pH 5.0, demonstrating the good O_2_ generation ability of HMP. These results suggested that HMP owned the multi-functional properties of ·OH and O_2_ generation, and GSH consumption, paving the way for enhanced anti-GBM efficiency.

Then, an infrared thermal camera was used to evaluate the photothermal behavior via monitoring the temperature change of HMP nanoparticles under 808-nm laser irradiation. As shown in Fig. S10, HMP nanoparticles displayed a time- and concentration-dependent temperature increase behavior under 808-nm laser irradiation than H_2_O, suggesting their efficient photothermal behavior. Moreover, the temperature increase of HMP nanoparticles was also dependent on the laser power density (Fig. S11). Subsequently, photothermal stability of HMP was investigated after five on/off cycles of laser irradiation. As shown in Fig. S12, HMP nanoparticles maintained similar temperature increment upon repeated laser irradiation, exhibiting excellent photothermal stability. The efficient photothermal conversion of HMP nanoparticles facilitates the hyperthermia-induced BBB penetration [66].

Collectively, we successfully developed a nanoplatform capable with multi-responsive properties including ·OH production, GSH consumption and O_2_ generation, as well as excellent photothermal conversion ability, providing powerful supports for the following study of overcoming the drug resistance of TMZ.

### In vitro drug loading, drug release, cellular uptake and multifunctional activity evaluation

**Fig. 3:**
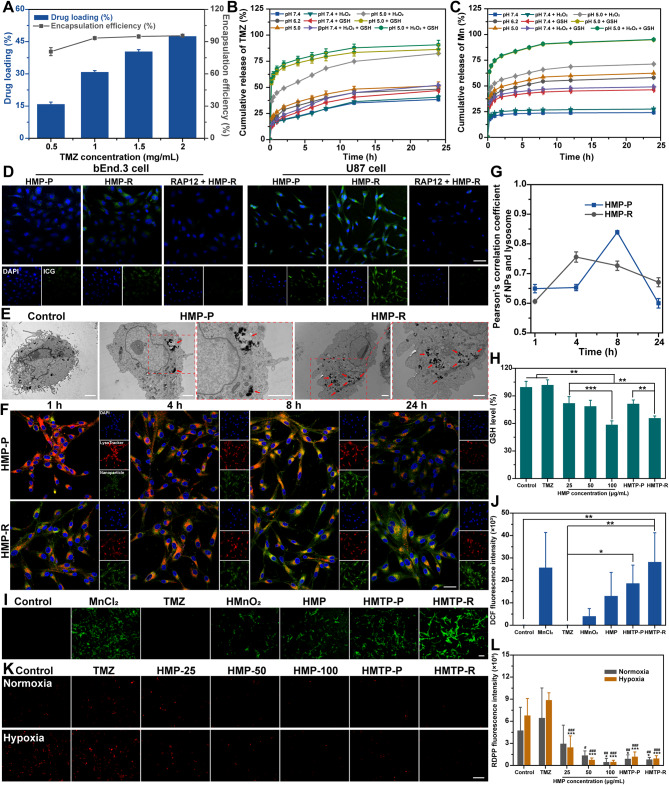
In vitro drug loading, drug release, cellular uptake and TME modulation ability evaluation. (**A**) Drug loading and encapsulation efficiency of TMZ in HMTP added with various amount of TMZ. Cumulative release of (**B**) TMZ and (**C**) Mn from HMTP at different conditions (pH 7.4, 6.2, or 5.2 with or without H_2_O_2_/GSH) (*n* = 3). (**D**) Cellular uptake of HMP-P or HMP-R in bEnd.3 and U87 cells. ICG (20 µg/mL) was used as an indicator of nanoparticles for CLSM imaging. Scale bar: 50 μm. (**E**) TEM images of U87 cells after PBS, HMP-P or HMP-R treatment. Scale bar: 2 μm. Red arrows indicate the uptake of nanoparticles. (**F**) In vitro endo/lysosomal escape capability of HMP-P or HMP-R in U87 cells. Scale bar: 30 μm. (**G**) Pearson’s correlation coefficient analysis of co-localization between green and red fluorescence by ImageJ (*n* = 3). (**H**) Relative intracellular GSH levels after incubated with PBS, TMZ, HMTP-P, HMTP-R or HMP at different concentrations. The data are presented as mean ± SD (*n* = 3). ^**^P < 0.01, ^***^P < 0.001. (**I**) Intracellular ROS generation was monitored by ROS probe (DCFH-DA) of U87 cells after PBS, MnCl_2_, TMZ, HMnO_2_, HMP, HMTP-P or HMTP-R treatment. Scale bar: 100 μm. (**J**) Corresponding quantitative results of DCF fluorescence intensity (*n* = 3). ^*^P < 0.05, ^**^P < 0.01. (**K**) Intracellular O_2_ generation was monitored via O_2_ sensor (RDPP) in U87 cells under normal or hypoxic condition after PBS, TMZ, HMTP-P, HMTP-R or HMP with various concentrations (25, 50, 100 µg/mL). Scale bar: 100 μm. (**L**) Corresponding quantitative of RDPP fluorescence intensity (*n* = 3). ^**^P < 0.01 vs. control, ^***^P < 0.001 vs. control, ^#^*P* < 0.05 vs. TMZ, ^##^*P* < 0.01 vs. TMZ, ^###^*P* < 0.001 vs. TMZ. The statistical significance was calculated via one-way ANOVA Tukey’s multiple comparisons test

For drug loading, TMZ at varied concentration was added to equal amount of HMnO_2_, the DL (%) and EE (%) increased with the increasing TMZ concentration, and reached a plateau of 47.74 ± 0.51% and 95.49 ± 1.02% at 2 mg/mL, respectively (Fig. 3A). And the Mn content in HMTP was about 35.24 ± 6.35%. Next, we studied the TME-triggered drug release of TMZ and Mn^2+^ from HMTP measured by UV-Vis spectroscopy and ICP, respectively (Fig. 3B and C). Compared to the slow drug release rate of HMP at normal physiological environment, the cumulative release of both TMZ and Mn^2+^ was found to be increased in the mild acidic condition (pH 6.2), and the released drugs were further enhanced in the endosome or lysosome environment (pH 5.0). Moreover, 90.73 ± 4.34% of TMZ and 95.14 ± 0.52% of Mn^2+^ were released in the presence of H_2_O_2_ and GSH at 24 h, due to the H_2_O_2_/GSH triggered decomposition of HMnO_2_ in HMP nanoparticles. The similar accelerated release behavior could be observed in the pH 7.4 environment with the addition of H_2_O_2_ and GSH. These results showed that HMP has high drug loading and encapsulation efficiency of TMZ, and the released curves of TMZ and Mn^2+^ were presented pH/H_2_O_2_/GSH-triggered release behavior.

Owing to the overexpression of LRP1 in BBB and GBM cells, RAP12-modified nanoparticles could be effectively taken up by brain microvascular endothelial cells and GBM cells via RMT [67]. Cellular uptake behavior was evaluated in bEnd.3 and U87 cells. As displayed in Fig. 3D, compared to the non-targeting group, higher fluorescence intensity could be observed both in bEnd.3 and U87 cells after incubated with HMP-R for 4 h, and displayed time-dependent fluorescence increase behavior (Figs. S13 and S14). In order to confirm the internalization mechanism of HMP-R nanoparticles, bEnd.3 and U87 cells were pre-treated with free RAP12 at a concentration of 200 µg/mL for 0.5 h, and CLSM was utilized to investigate the change of fluorescence intensity. It was shown that after pre-treatment of free RAP12, the cellular uptake of HMP-R was decreased within 2 h, and even no obvious fluorescence could be observed at 4 h. Flow cytometry results of the bEnd.3 and U87 cells also confirmed the similar phenomenon (Figs. S13C and S14C). Besides, cellular TEM images further showed that more nanoparticles were observed in U87 cells incubated with HMP-R compared to HMP-P (Fig. 3E). As RAP12 peptide could selectivey bind to the LRP1 receptor, the above results implied that the cellular uptake of HMP-R mainly depends on RAP12-mediated RMT. The endo/lysosomal escape of the nanoparticles is a requirement for nanoparticles functionality. Hence, we studied the in vitro endo/lysosomal escape property of HMP-P and HMP-R in U87 cells via CLSM. As shown in Fig. 3F-G, after co-incubation for 4 h, the HMP-R showed high colocalization with endo/lysosomes, while 8 h-incubation of HMP-P. Interestingly, most of HMP-P or HMP-R and endo/lysosomes did not overlap after 24 h incubation, following by the decrease of Pearson’s correlation coefficient index, suggesting that HMP-P and HMP-R could escape from endo/lysosomes over time.

After that, the in vitro modulation of TME that associated with drug resistance was detected by ROS, GSH and O_2_ probes. Firstly, the intracellular GSH level also exhibited a concentration-dependent behavior in the U87 cells after HMP treatment. And HMTP-R showed higher GSH consumption than HMTP-P at the concentration of 50 µg/mL, which mainly is due to the enhanced cellular uptake after RAP12 modification (Fig. 3H). Then, as the DCFH-DA probe would be oxidized to dichlorofluorescein (DCF) by ROS which displayed green fluorescence for observation, DCFH-DA therefore was utilized for intracellular ROS level detection. As illustrated in Fig. 3I and J, nearly no green fluorescence could be observed from the control and TMZ groups. While U87 cells treated with MnCl_2_, HMnO_2_, HMP, HMTP-P and HMTP-R showed discernable green fluorescence. Notably, stronger green fluorescence intensity of U87 cells after HMTP-R treatment was similar to that after free MnCl_2_ treatment as the same Mn concentration, indicating that HMTP-R could effectively induce intracellular oxidative stress. In addition, intracellular O_2_ generation was investigated using RDPP indicator in U87 cells under normal or hypoxic condition after catalyzed by HMP, HMTP-P and HMTP-R. An increased red fluorescence was observed both in untreated cells and the cells after TMZ treatment in hypoxic environment (Figs. 3K and L and S15). However, U87 cells displayed decreased red fluorescence in a concentration-dependent behavior after HMP treatment both under normal or hypoxic condition, which demonstrated that HMP could catalyze the intracellular H_2_O_2_ to generate O_2_ to relieve hypoxia situation in cancer cells. In vitro studies also showed that, HMP exhibited enhanced ROS generation, O_2_ production and GSH consumption properties, further confirming the TME modulation ability of HMP.

### In vitro BBB crossing, deep penetration and therapeutic effect

**Fig. 4:**
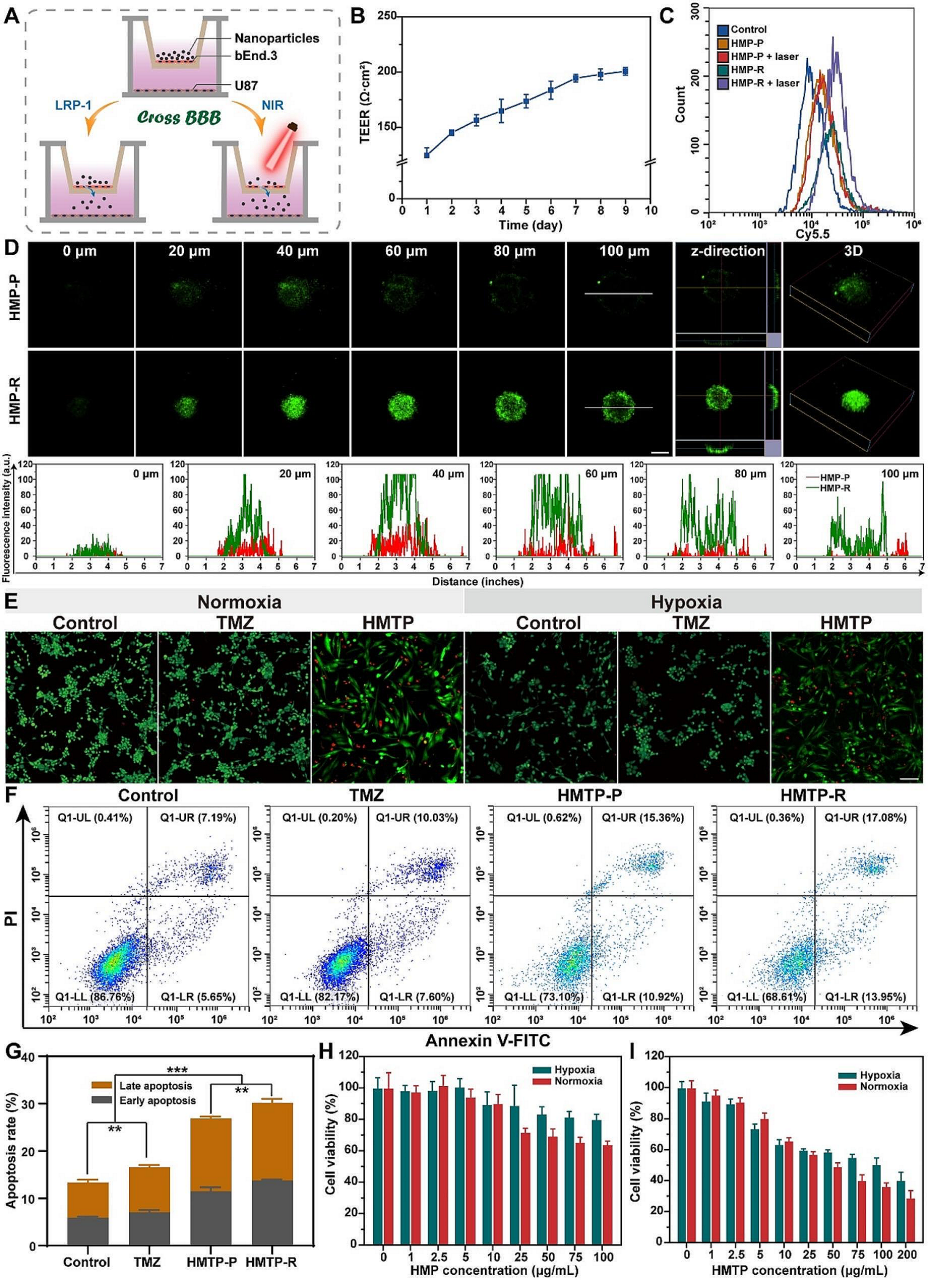
In vitro BBB transport efficiency and anti-tumor effect evaluation. (**A**) Schematic illustration of the establishment of in vitro BBB transwell model. (**B**) The dynamic TEER values of bEnd.3 cells monolayers within 9 days (*n* = 3). (**C**) Quantitative flow cytometric analysis showing the uptake of nanoparticles by U87 cells after PBS, HMP-P, and HMP-R with or without laser irradiation treatment. (**D**) Representative multilevel scan and dynamic intensity of ICG fluorescence of HMP-P and HMP-R in 3D tumor spheroids after incubation for 8 h. Scale bar: 200 μm. (**E**) Live/dead staining images of U87 cells after different treatment. Scale bar: 100 μm. (**F**) Cell apoptosis by flow cytometric analysis and (**G**) its quantification after treatment with PBS, TMZ, HMTP-P and HMTP-R. The data are presented as mean ± SD (*n* = 3). ^**^P < 0.01, ^***^P < 0.001. Cell viability of U87 cells after (**H**) HMP or (**I**) HMTP treatment at various concentrations under hypoxia or normoxia environment (*n* = 5). The statistical significance was calculated via one-way ANOVA Tukey’s multiple comparisons test

Owning to the favorable cellular uptake efficiency of HMP-R in both the bEnd.3 and U87 cells, an in vitro BBB model was employed to evaluate the ability of HMP-R nanoparticles to traverse the BBB and cellular uptake efficiency of GBM cells, as well as the potency of NIR to modulate the permeability of BBB. A transwell BBB model was constructed with a bEnd.3 cells monolayer grown on the upper chamber, and U87 cells grown on the lower chamber, as illustrated in Fig. 4A. Firstly, we evaluated the intracellular photothermal conversion in bEnd.3 cells. The temperature of cells exhibited a concentration- and laser power density-dependent manner under laser irradiation for 10 min, revealing the possibility of BBB permeability regulation by NIR irradiation (Fig. S16). After that, the BBB model was constructed for 9 days until the TEER above 200 Ω·cm^2^ (Fig. 4B). After the treatments, the U87 cells on the lower chamber were collected for flow cytometry assay to evaluate the BBB permeability. Results showed that the transport ratio of RAP12 targeting peptide-modified nanoparticles (17.19%) was higher than that of non-targeting peptide groups (6.74%) (Fig. 4C and S17). After NIR irradiation, the transport ratio of HMP-R was further enhanced to 26.39%, indicating that the combination of LRP-mediated transcytosis and NIR-modulated BBB permeability could efficiently improve the BBB crossing ability of HMP. Notably, the TEER did not obviously change after HMP treatment for 24 h, suggesting that HMP nanoparticles would not cause any damage to the tight junctions of BBB (Fig. S18). However, NIR irradiation would lower the TEER of bEnd.3 monolayer in the presence of HMP-P or HMP-R, and the TEER level could return back to normal after removing the irradiation, suggesting a reversable strategy for permeability regulation. These results demonstrated that the “LRP-NIR” dual-targeted strategy could effectively enhance the ability of HMP-R nanoparticles to traverse the BBB, while cause nearly no damage to BBB.

Next, we established a 3D tumor spheroid model to further evaluate the deep penetration of HMP-P and HMP-R. After incubation with HMP-P or HMP-R for 8 h, the fluorescence images at different tumor penetration depths (0–100 μm) were displayed in Fig. 4D. The results displayed that the penetration depth and the fluorescence intensity in the tumor spheroids after HMP-R treatment were deeper and stronger than those in the HMP-P, indicating that RAP12 peptide-targeting modification promoted the tumor deep penetration of nanoparticles.

Finally, the Live/dead assay, cell apoptosis and cytotoxicity kit assay were used to evaluate the in vitro U87 cells viability after treatment exposure. Firstly, the cells after different treatment under both normoxia and hypoxic condition were co-stained with Calcein-AM and PI for CLSM imaging. The green fluorescence and red fluorescence represented live cells and dead cells, respectively. As displayed in Fig. 4E and Fig. S19, HMTP could cause more damages to U87 cells both under normoxia and hypoxic conditions. Then, the cell apoptosis ratio after various treatments was analyzed by co-stained with Annexin V-FITC and PI, followed by flow cytometry test. The results showed that the percentage of apoptosis cells after treated with HMTP-R was 30.14 ± 0.91%, which was higher than that of non-targeting peptide-modified groups of HMTP-P (26.87 ± 0.56%), TMZ (16.58 ± 0.91%) and PBS (13.37 ± 0.66%) (Fig. 4F and G).

Then, the cytotoxicity of HMP and HMTP in U87 cells was evaluated via CCK-8. The results showed that with the assistance of O_2_, HMTP led to 70.11 ± 5.03% cell death at 200 µg/mL under normoxic condition, which was higher than that of hypoxic condition, suggesting that self-generation of O_2_ could relieve the hypoxia of TME, and further enhance the chemotherapy of TMZ (Fig. S20). Then, the cytotoxicity analysis of HMP and HMTP was conducted. As indicated, cell viability of both HMP and HMTP was concentration-dependent under both normoxic and hypoxic condition (Fig. 4H and I). Cells treated with HMTP showed lower cell viability (36.38 ± 2.04%) than that of HMP group (63.98 ± 2.12%) at the concentration of 100 µg/mL, suggesting the effectiveness of synergistic therapy of HMTP. These results implied that, hypoxia environment would hamper the anti-tumor effect, reminding that alleviating hypoxia environment would contribute to overcoming drug resistance of chemotherapy, further improving the therapeutic effect.

**Fig. 5:**
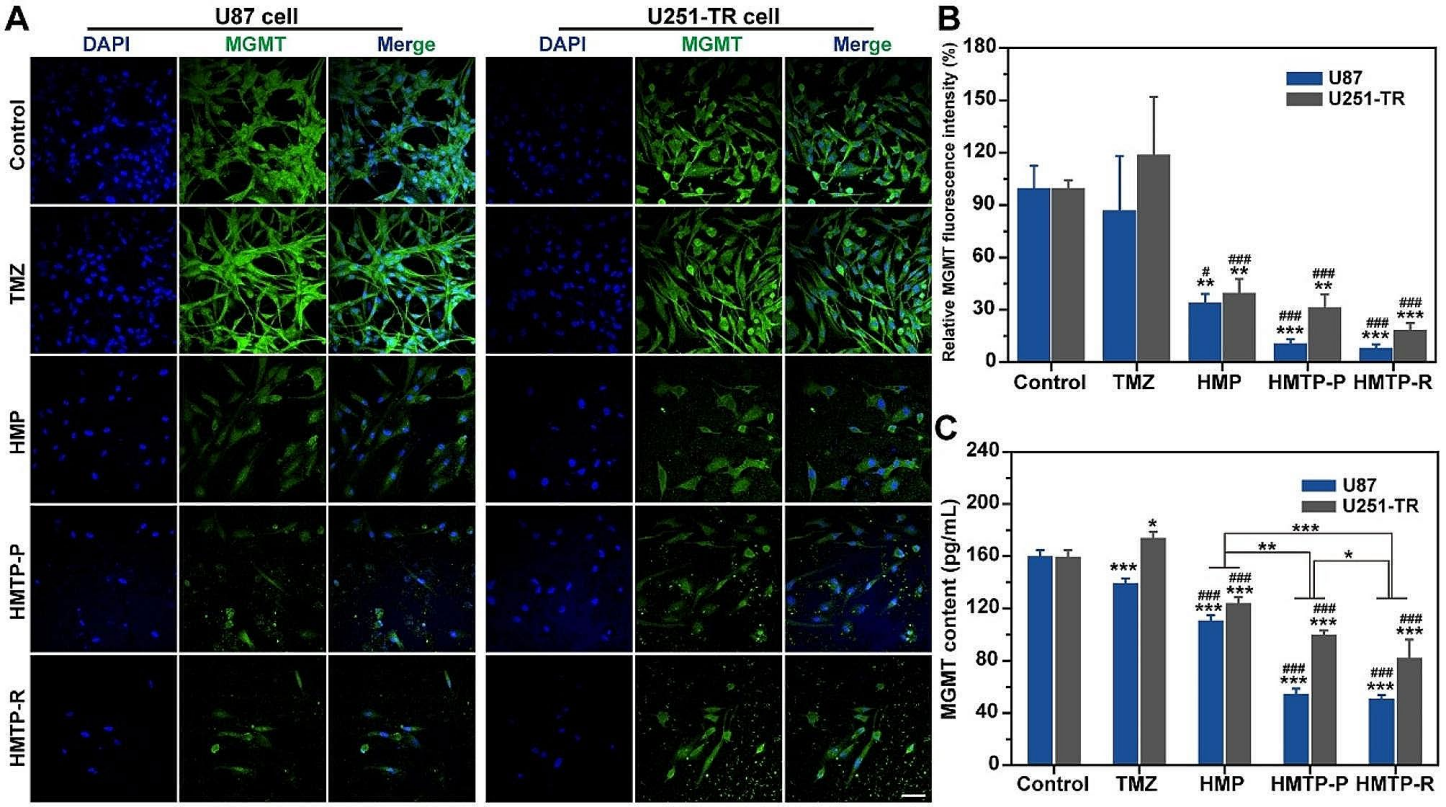
Evaluation of MGMT expression. (**A**) CLSM images of MGMT fluorescence, (**B**) relative MGMT fluorescence intensity and (**C**) MGMT content on U87 and U251-TR cells after incubated with PBS, TMZ, HMP, HMTP-P or HMTP-R for 72 h (*n* = 3). Scale bar: 50 μm. ^*^P < 0.05 vs. control, ^**^P < 0.01 vs. control, ^***^P < 0.001 vs. control, ^#^*P* < 0.05 vs. TMZ, ^###^*P* < 0.001 vs. TMZ. The data are presented as mean ± SD. The statistical significance was calculated via one-way ANOVA Tukey’s multiple comparisons test

MGMT is a DNA repairing protein which could efficiently remove the DNA lesions generated by TMZ, and contribute to refractory TMZ resistance [12]. Relieving hypoxia environment and promoting ROS generation would down-regulate the drug resistance of TMZ. Accordingly, immunofluorescence staining showed that the MGMT protein expression in U87 cells decreased after incubated with HMP, HMTP-P and HMTP-R compared to control and TMZ treatment, and HMTP-R induced the highest reduction of MGMT expression (8.55 ± 1.62%) (Fig. 5A and B). Similar results could be observed for HMTP-R in U251-TR cells. Meanwhile, MGMT content in U87 and U251-TR cells were also detected via Elisa assay, which was coincidence with the results of immunofluorescence staining assay (Fig. 5C). Both immunofluorescence staining and Elisa assay confirmed that HMP, HMTP-P and HMTP-R could efficiently decrease the MGMT expression on whether U87 or U251-TR cells, further downregulating the drug resistance of TMZ.

### In vivo imaging, tissue biodistribution and pharmacokinetics

**Fig. 6:**
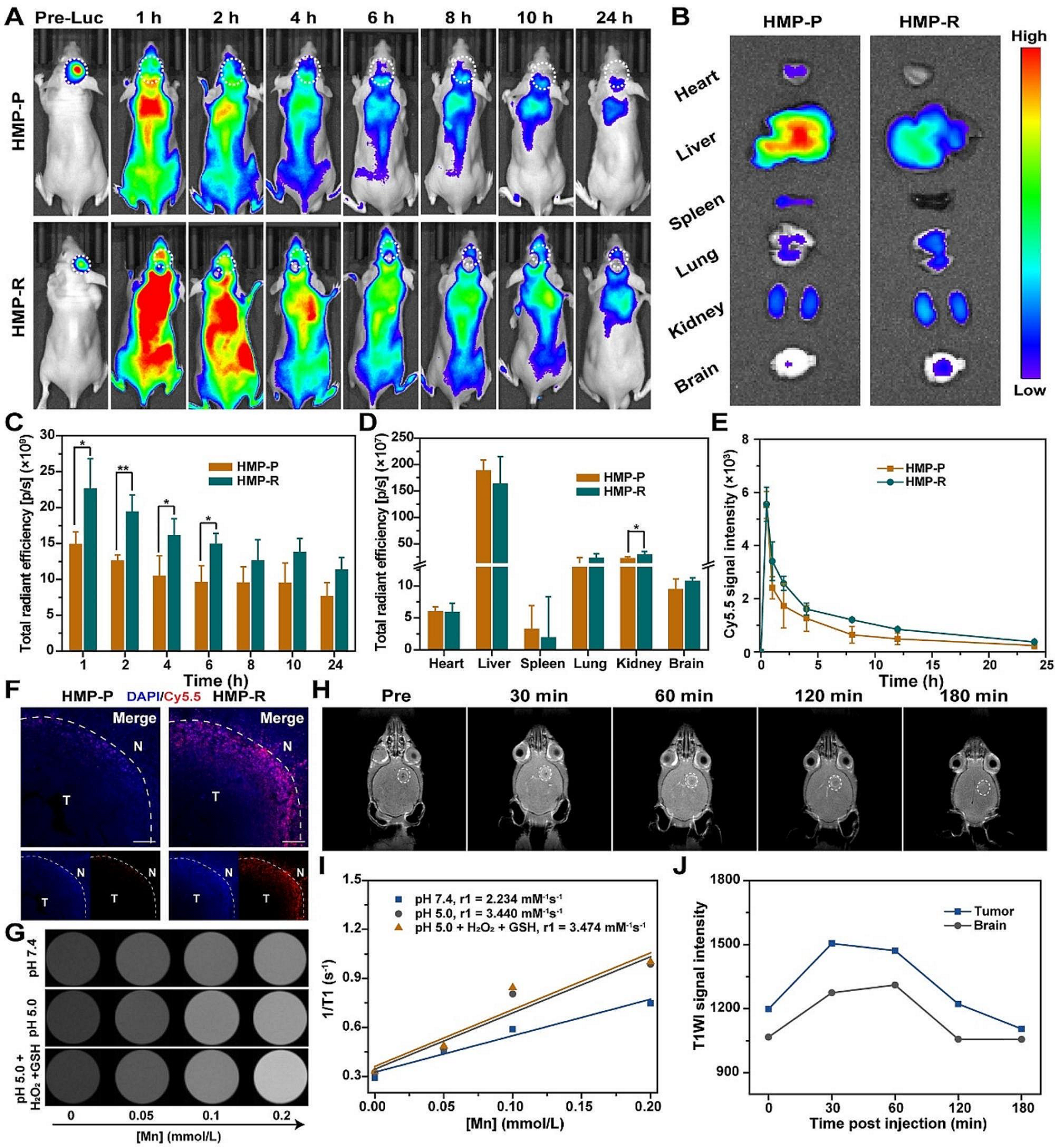
In vivo brain tumor targeting and distribution of HMP-R. (**A**) Time-dependent in vivo fluorescence imaging of U87-tumor-bearing mice after injection of HMP-P and HMP-R. (**B**) Ex vivo fluorescence images of major organs collected at 24 h post-injection. (**C**) Total radiant efficiency of the tumor sites (*n* = 3). (**D**) Total radiant efficiency of the ex vivo major organs (*n* = 3). (**E**) Pharmacokinetics profiles of HMP-P and HMP-R (*n* = 5). (**F**) The distribution of Cy5.5-labeled nanoparticles in the brain tissues observed via CLSM. (**G**) T1-weighted images of HMP under different conditions. (**H**) In vivo T1-weighted MRI images of U87-tumor-bearing mice after HMP-R injection. (**I**) Corresponding r1 value of HMP at various pH solution. (**J**) Corresponding T1WI signal intensity of the tumor and contralateral normal brain region after HMP-R injection. The data are presented as mean ± SD. *P < 0.05, **P < 0.01. The statistical significance was calculated via one-way ANOVA Tukey’s multiple comparisons test

To construct U87-Luc cells, we transfected U87 cells with lentiviral vectors carrying Luc, and verified successful transfection by IVIS imaging, followed via purification by puromycin to gain stable and passable U87-Luc cells (Fig. S21). To evaluate the in vivo BBB and GBM targeting ability of RAP12 peptide, we compared the BBB and GBM penetrating capability of HMP-P and HMP-R nanoparticles labeled by fluorescent tag Cy5.5 in U87-Luc tumor-bearing mice. Cy5.5-labeled HMP-R could migrate into the brain after injection, and its fluorescence signal was stronger than non-targeting peptide modification in the brain (Figs. 6A and C, and S22A-B). Major organs of treated mice were collected at 24 h post-injection for ex vivo imaging. The fluorescence signal in the brain after HMP-R injection was higher than the HMP-P group, and significantly higher at 2 h (*P* < 0.01), indicating that RAP12 modification could effectively enhance the accumulation of nanoparticles in the GBM (Fig. 6B and D). To investigate the in vivo pharmacokinetics of HMP-P or HMP-R, Cy5.5 was used to label the nanoparticles for fluorescence signal intensity analysis of blood plasma levels. Results in Fig. 6E showed that HMP-P or HMP-R treatment could prolong the bloodstream circulation with half-life of 7.80 ± 4.11 h and 8.28 ± 1.19 h, respectively, which was higher than free TMZ of less than 10 min according to previous reports [68]. The immunofluorescence imaging showed that higher fluorescence signals of Cy5.5 were observed in the GBM regions of HMP-R, particularly in striatum, further demonstrating the GBM-targeting ability of RAP12 in vivo (Figs. 6F, S22 and S23). It is worth mentioning that the Cy5.5 fluorescence of non-targeting peptide modified nanoparticles (HMP-P) in the brain was mainly induced by the destruction of BBB under the deterioration of GBM. In addition, we verified the NIR-triggered BBB opening in vivo. As shown in Fig. S24, the treatment of 808-nm laser irradiation could enhance the fluorescence accumulation in the brain, and prolong its retention time in the brain, implying that the “LRP-NIR” dual-targeted strategy would improve the BBB permeability and GBM targeting properties of the nanomaterials. After administration, a large amount of nanoparticles would accumulate in the reticuloendothelial system (RES) organs, including liver, lung and spleen, followed by metabolized and excreted out of the body via kidney. Only a tiny amount of non-targeted nanoparticles could reach the brain, which is thwarted by physical barriers in the brain of BBB and intrinsically malignant nature of BBTB in GBM [69]. Both HMP-P and HMP-R nanoparticles might be eliminated by liver and kidney over time (Fig. S25).

Next, we investigated the TME-activated MRI of HMP-R nanoparticles in vitro and in vivo. As shown in Fig. 6G, a concentration-dependent brightness was monitored in pH 5.0 solution added with 1 mM H_2_O_2_ and 10 mM GSH. And the r1 value at pH 5.0 with or without H_2_O_2_ and GSH was higher than that at pH 7.4 (Fig. 6I). Then the brain-targeting performance of HMP-R of tumor was evaluated in GBM-tumor bearing mice. As displayed in Fig. 6H and J, after mice received intravenous injection with HMP-R, the T1WI signal intensity of both tumor and brain increased from 30 min, and reached the peak at 1 h, followed by a gradual decrease, which was in coincidence with the in vivo imaging results. Both in vitro and in vivo MRI results demonstrated that HMP-R could be utilized as a potential contrast agent triggered by TME for brain tumor detection.

### In vivo therapeutic efficacy and biosafety evaluation

**Fig. 7:**
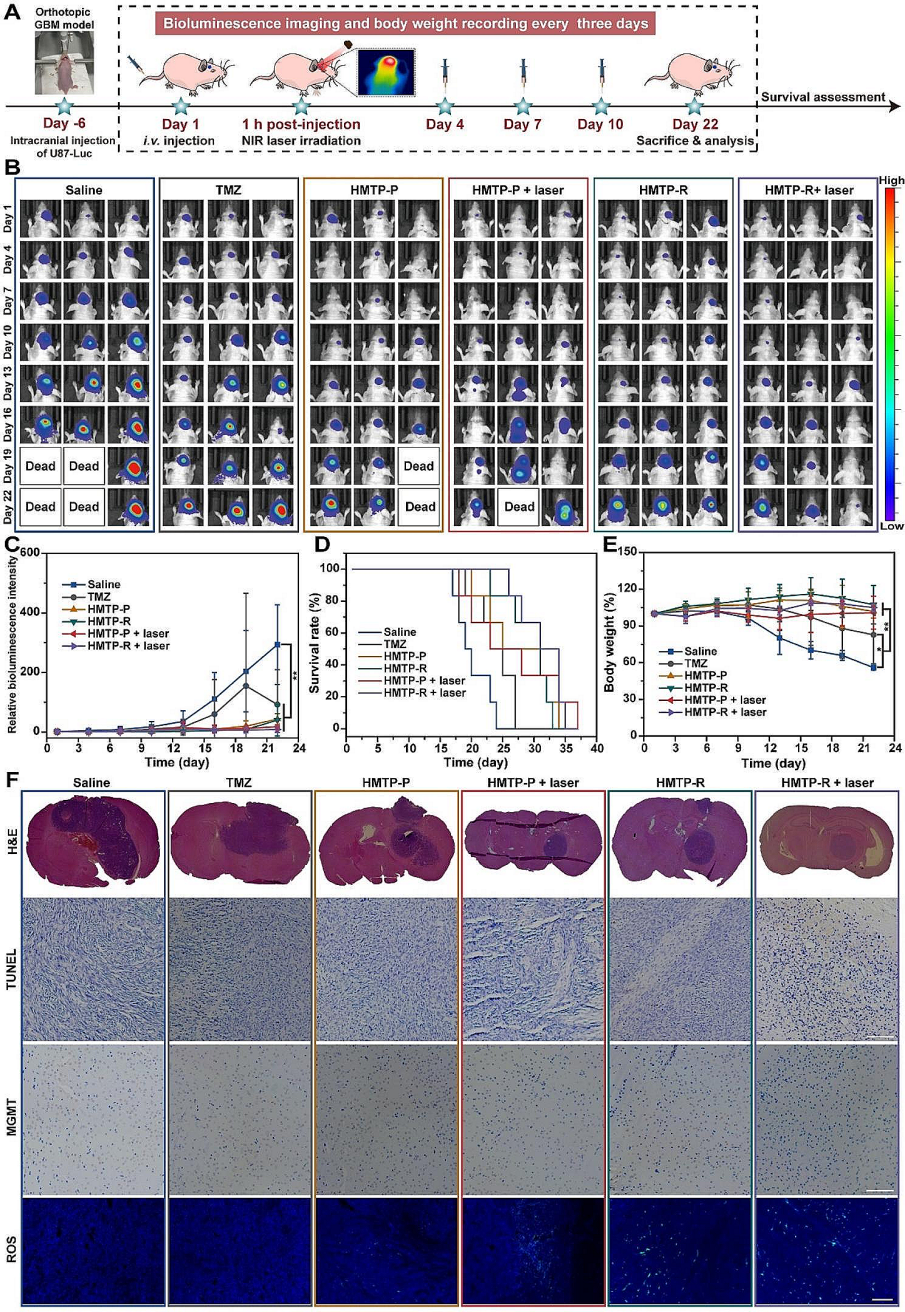
In vivo therapeutic effect in nude mice bearing orthotopic U87-Luc-tumor. (**A**) Schematic illustration of the model establusion and therapeutic protocol. (**B**) Representative in vivo bioluminescent images of GBM-bearing mice after treatment with Saline, TMZ, HMTP-P, HMTP-R with or without 808-nm laser irradiation. (**C**) Relative U87-Luc tumor luminescence levels of mice with different treatments (*n* = 6). (**D**) Survival ratio of mice after different treatments (*n* = 6). (**E**) Body weight changes of mice after different treatments (*n* = 6). The data are presented as mean ± SD. *P < 0.05, **P < 0.01. (**F**) Brain sections from mice on day 22 after different treatments stained with H&E, TUNEL, MGMT or ROS staining. Scale bar: 100 μm. The statistical significance was calculated via one-way ANOVA Tukey’s multiple comparisons test

The in vivo therapeutic efficacy of nanoparticles was investigated in orthotopic U87-Luc GBM-bearing nude mice, which were treated with saline, free TMZ, HMTP-P, HMTP-P + laser, HMTP-R, and HMTP-R + laser, respectively, at the same TMZ dosage of 2.5 mg/kg (Fig. 7A). The tumor growth of GBM was monitored by luciferin bioluminescence of U87-Luc cells under IVIS. During the 22-day treatment period, the tumor volume of saline group gradually increased over time, while that the mice receiving HMTP-P or HMTP-R treatment displayed a certain inhibitory effect on the GBM growth (Fig. 7B and C). Furthermore, after the laser irradiation application, both HMTP-P + laser and HMTP-R + laser group showed enhanced inhibition effect on tumor growth, indicating that the combination of chemotherapy and CDT exhibited satisfactory tumor inhibition. Dual-targeting ability could further improve the therapeutic efficacy. The survival rate of nude mice receiving HMTP-P + laser treatment significantly prolonged the survival time of GBM-bearing mice compared with saline group (*P* < 0.01) (Fig. 7D). It is worth noting that the body weight of tumor-bearing nude mice after HMTP-P or HMTP-R with or without laser irradiation treatment showed no significant changes during the treatment, while the mice treated with saline and TMZ treatment suffered an accelerated weight loss (Fig. 7E). The body weight profiles further confirmed the biosafety of our nanoparticles. H&E staining of the brains showed that the tumor of nude mice treated with HMTP-R + laser exhibited the highest tumor suppression, and with the smallest tumor area of brain compared to other treatment groups, which was in line with the above bioluminescence results. Besides, TUNEL assays showed the highest level of cell apoptosis of brain tumor treated with HMTP-R + laser. The level of MGMT was decreased after HMTP-R + laser treatment, supporting HMTP-R could inhibit the expression of MGMT and thereby increase DNA damage. Moreover, there was no apparent damage in H&E staining of major organs in all groups (Fig. S26). In addition, compared with other treatment groups, higher ROS fluorescence could be observed at the tumor tissue after mice injected with HMTP-R with laser irradiation, further demonstrating the excellent ROS production capacity of HMTP in vivo (Fig. 7F).

In order to further validate the in vivo biosafety of our treatment, we investigated the hemolysis ratio of HMP. As shown in Fig. S27, no detectable hemolysis was observed after various concentration of HMP treatment for 2 h, and the hemolysis ratio of only 2.37 ± 0.33% even at 500 µg/mL level, which was far lower than the permissible limit (5%), indicating the good hemocompatibility of HMP for intravenous administration. Meanwhile, open field test was performed after whole treatment period (Fig. S28). Heatmap and typical motion route for 15 min after various nanoparticles injection displayed similar travel patterns compared to the control group, and the total distance, average speed, as well as mean speed at the center, showed limited difference after the treatments. These results demonstrated that the exploratory and locomotor activities of mice were not influenced by the administration of nanoparticles. Furthermore, blood samples were collected after various treatments, and utilized for blood chemistry analysis. No obvious hepatorenal damage was observed of mice with various nanoparticles treatment after the therapeutic period (Fig. S29). Conclusively, HMTP-R has good biosafety, and would not cause significant toxic side effects.

## Conclusions

In summary, we developed a dual-targeted and TME-responsive drug delivery nanoplatform, TMZ@HMnO_2_@PDA-PEG-RAP12 (HMTP-R), by loading a chemotherapeutic agent TMZ in HMnO_2_ nanoparticles, and functionalized with PDA and the RAP12 targeting ligand for photothermal and RMT dual-targeted delivery. The obtained HMTP-R nanoparticles were capable of efficient drug loading, BBB/BBTB permeability, and multi-responsive properties for orthotopic GBM treatment. Once accumulated in the GBM tumor site, the HMTP-R nanoparticles could respond to the TME condition of mild acidicity, highly overexpressed GSH and H_2_O_2_, and release Mn^2+^, O_2_ and TMZ. The hypoxia alleviation via O_2_ production and redox balance disruption including GSH depletion and ·OH generation, could further reduce the expression of MGMT and overcome the drug resistance of TMZ. The released Mn^2+^ would catalyze endogenous H_2_O_2_ into ·OH via Fenton-like reaction, to further improve the in vivo anti-tumor effect of chemotherapy/chemodynamic therapy against GBM. This nanoplatform may serve as a powerful tool dealing with the low efficiency of drug delivery, and provide a potential therapeutic strategy for GBM treatment.

### Electronic supplementary material

Below is the link to the electronic supplementary material.

Supplementary Material 1

## Data Availability

All data generated or analyzed during this study are included in this published article.
