# Supplementary material for: Dual-targeted delivery of temozolomide by multi-responsive nanoplatform via tumor microenvironment modulation for overcoming drug resistance to treat glioblastoma
Source: J Nanobiotechnology. 2024 May 17;22:264. doi: 10.1186/s12951-024-02531-3 (PMC11100207; doi:10.1186/s12951-024-02531-3)
Supplement: Supplementary file 1 — Supplementary Material 1 [file 12951_2024_2531_MOESM1_ESM.docx]

Supporting Information

Dual-targeted delivery of temozolomide by multi-responsive nanoplatform via tumor microenvironment modulation for overcoming drug resistance to treat glioblastoma

Xiaojie Chen^1†^, Yuyi Zheng^1†^, Qi Zhang^1^, Qi Chen^1^, Zhong Chen^1, *^, Di Wu^1, *^

^1^ Key Laboratory of Neuropharmacology and Translational Medicine of Zhejiang Province, The First Affiliated Hospital and School of Pharmaceutical Sciences, Zhejiang Chinese Medical University, Hangzhou 310053, China.

*Corresponding authors. Emails: [wudichem@zju.edu.cn](mailto:wudichem@zju.edu.cn); chenzhong@zju.edu.cn;

Contributing authors: chenxiaojie0902@163.com; [zyy1265422764@163.com](mailto:zyy1265422764@163.com); zhangqihyun@163.com; cq-chem@zju.edu.cn

†These authors contributed equally to this work.


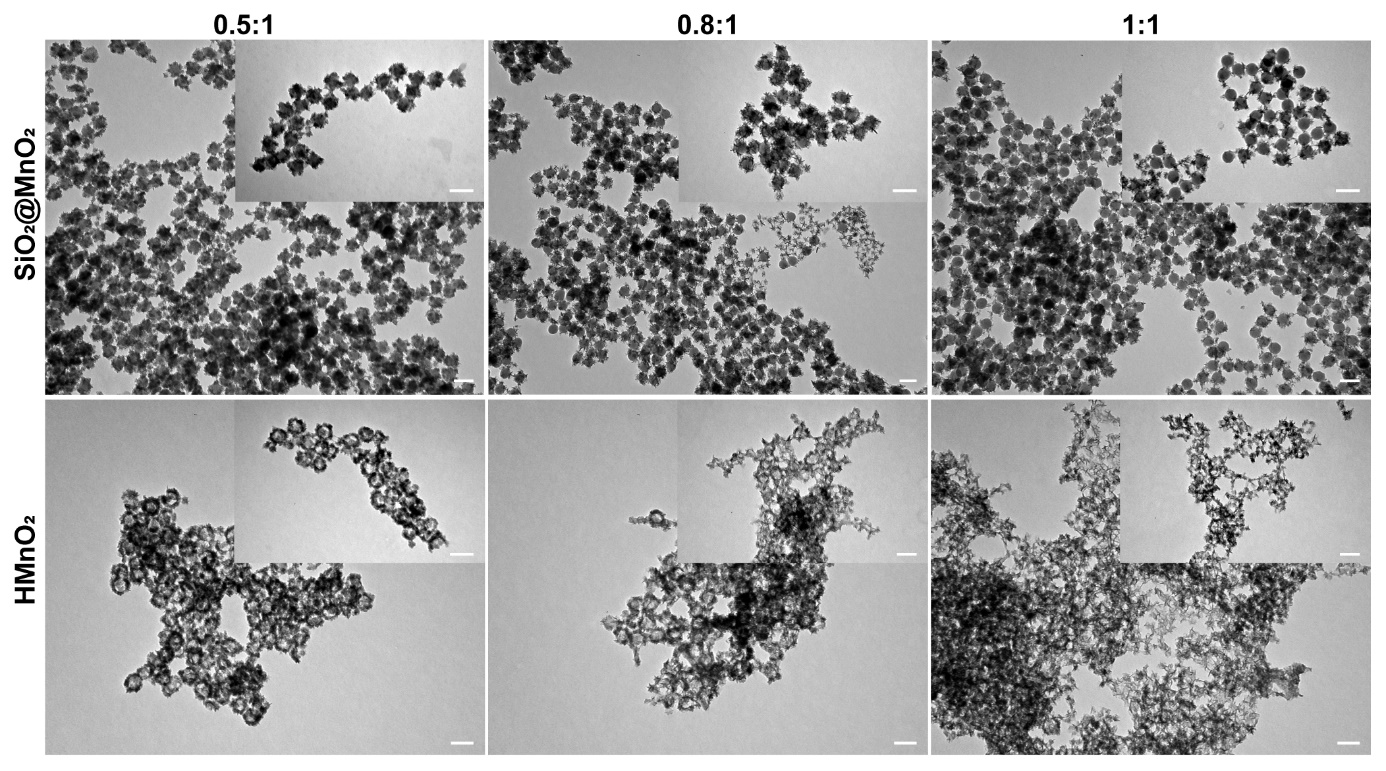


**Fig. S1** TEM images of SiO_2_@MnO_2_ and HMnO_2_ with various ratio of SiO_2_ and KMnO_4_. Scale bar: 100 nm.


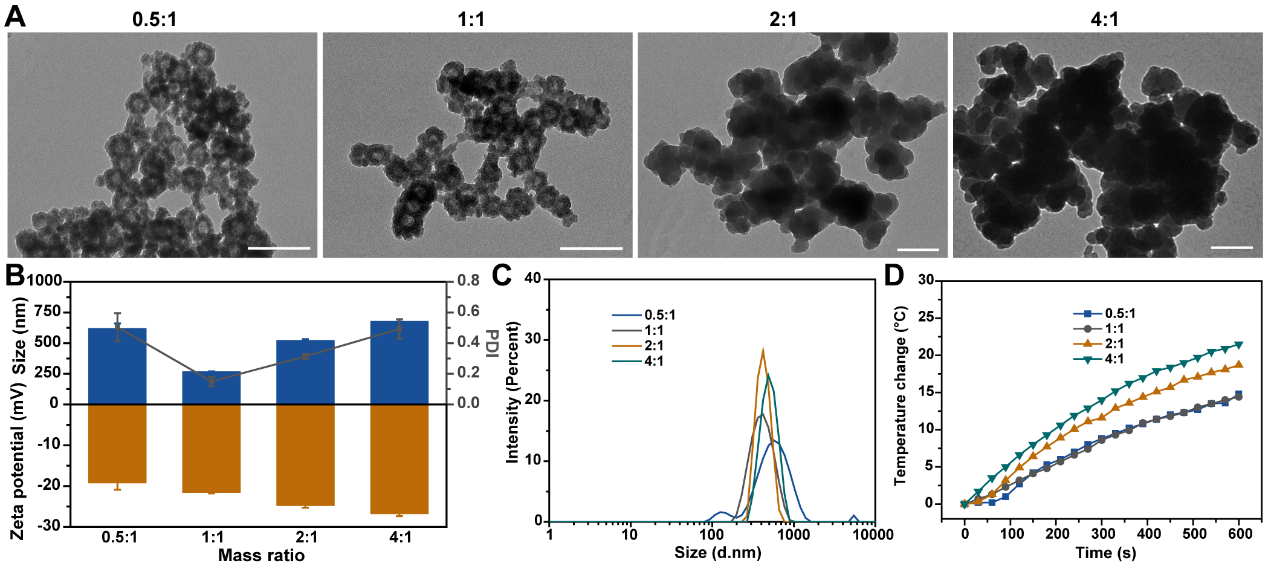


**Fig. S2** (A) TEM images of HMP at various mass ratio of DA and HMnO_2_. Scale bar: 200 nm. (B) Hydrodynamic diameter, PDI and zeta potential of HMP with various mass ratio (n = 3). (C) Size distribution of HMP with various mass ratio. (D) Temperature change curve of HMP (100 μg/mL) with various mass ratio.


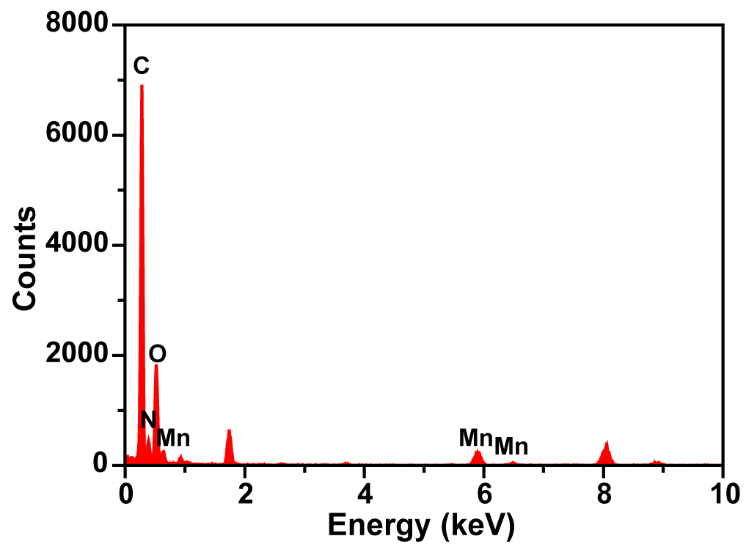


**Fig. S3** EDS spectrum of HMP.


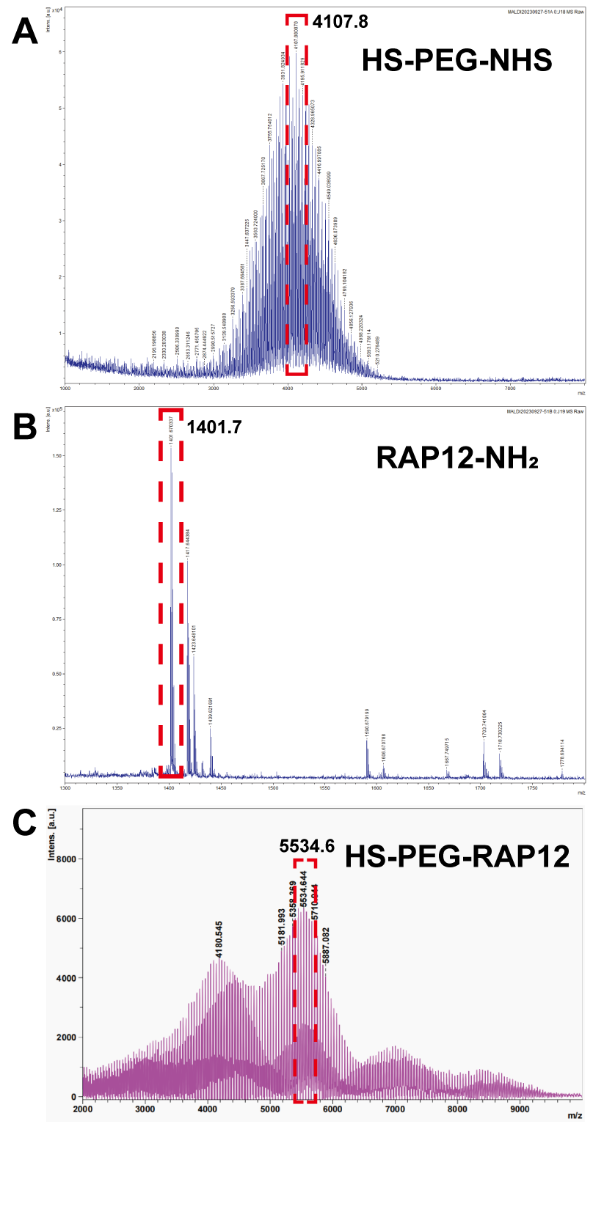


**Fig. S4** Matrix-assisted laser desorption/ionization time-of-flight mass spectra (MALDI-TOF MS) of (A) HS-PEG-NHS, (B) RAP12-NH_2_ and (C) HS-PEG-RAP12. The molecular weight of HS-PEG-NHS, RAP12-NH_2_, and HS-PEG-RAP12 was determined to be ~4100, ~1400, and ~5500 Da, respectively.

***
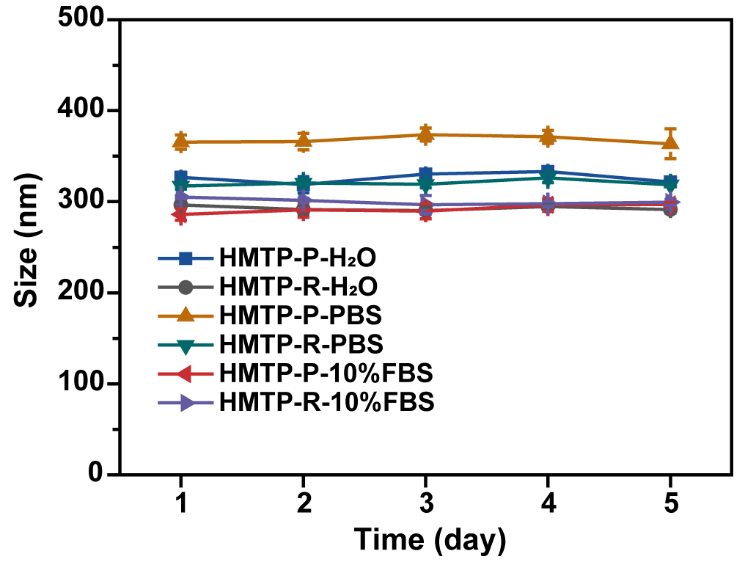
***

**Fig. S5** The stability evaluation of HMTP-P and HMTP-R in various medium (n = 3).


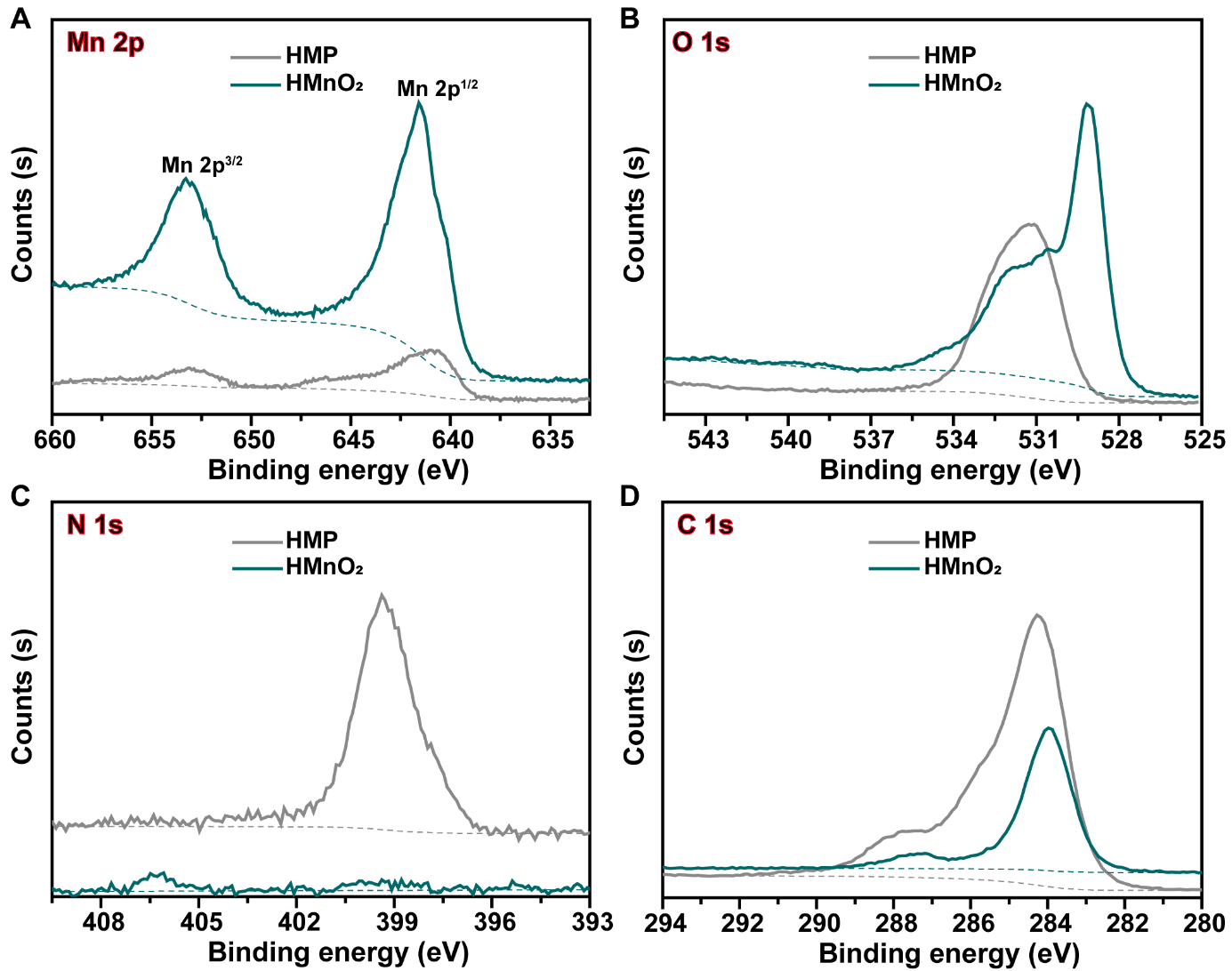


**Fig. S6** XPS analysis of HMnO_2_ and HMP nanoparticles. Survey spectrum of (A) Mn 2p, (B) O 1s, (C) N 1s, and (D) C 1s.


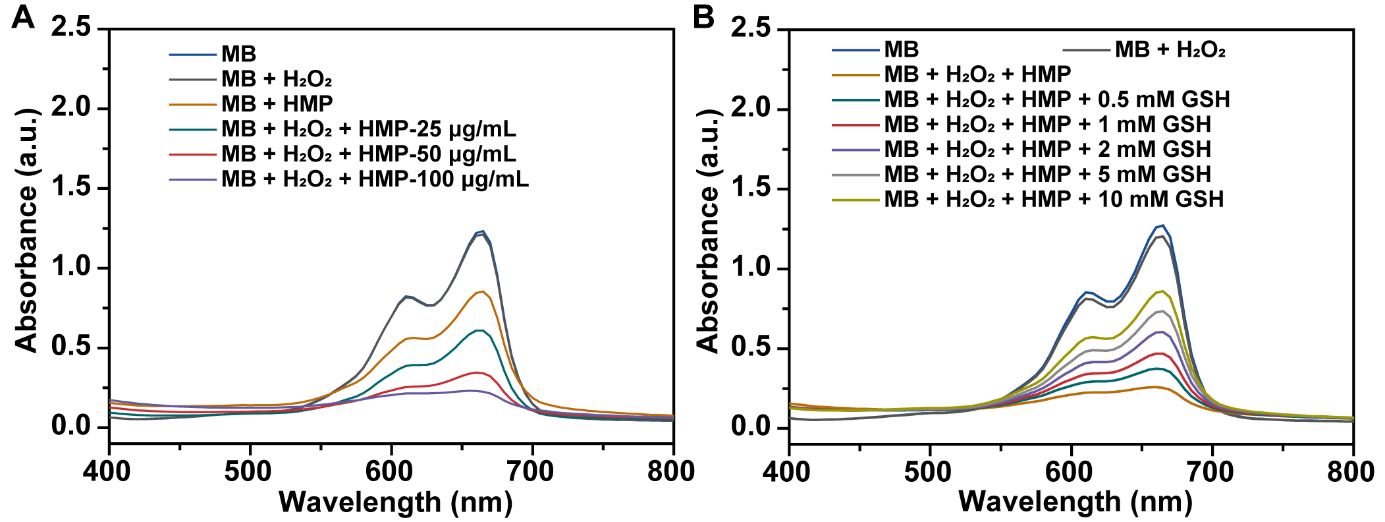


**Fig. S7** (A) MB degradation by various concentration of HMP in the presence of 10 mM H_2_O_2_, which was corresponding to Fig. 1G. (B) MB degradation by HMP (100 μg/mL) under different GSH concentrations (0-10 mM), which was corresponding to Fig.1H.


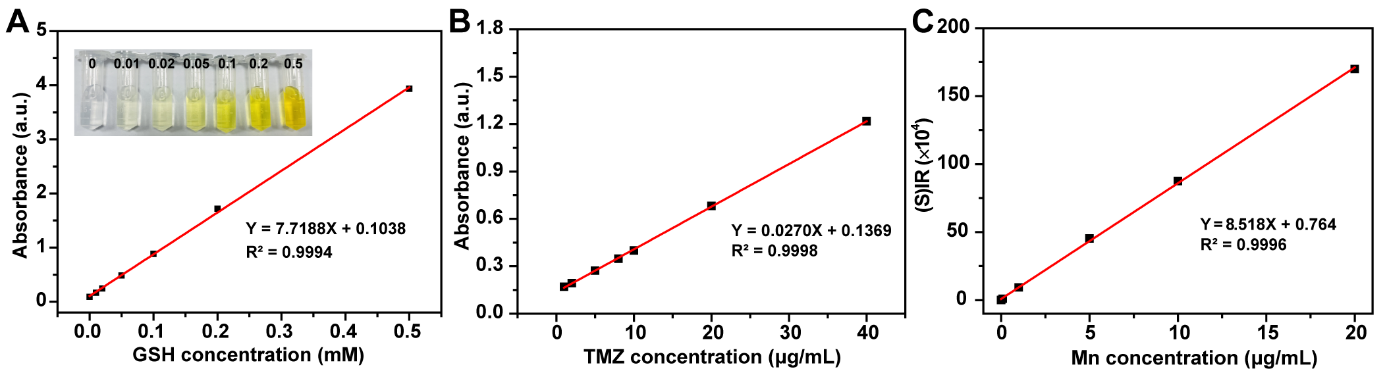


**Fig. S8** (A) Standard curve of absorbance of DTNB after treated with different GSH concentrations (0-0.5 mM), the absorbance was measured at 412 nm. (B) Standard curve of absorbance of TMZ, the absorbance was measured at 328 nm. (C) Standard curve of (S)IR of Mn measured via ICP.


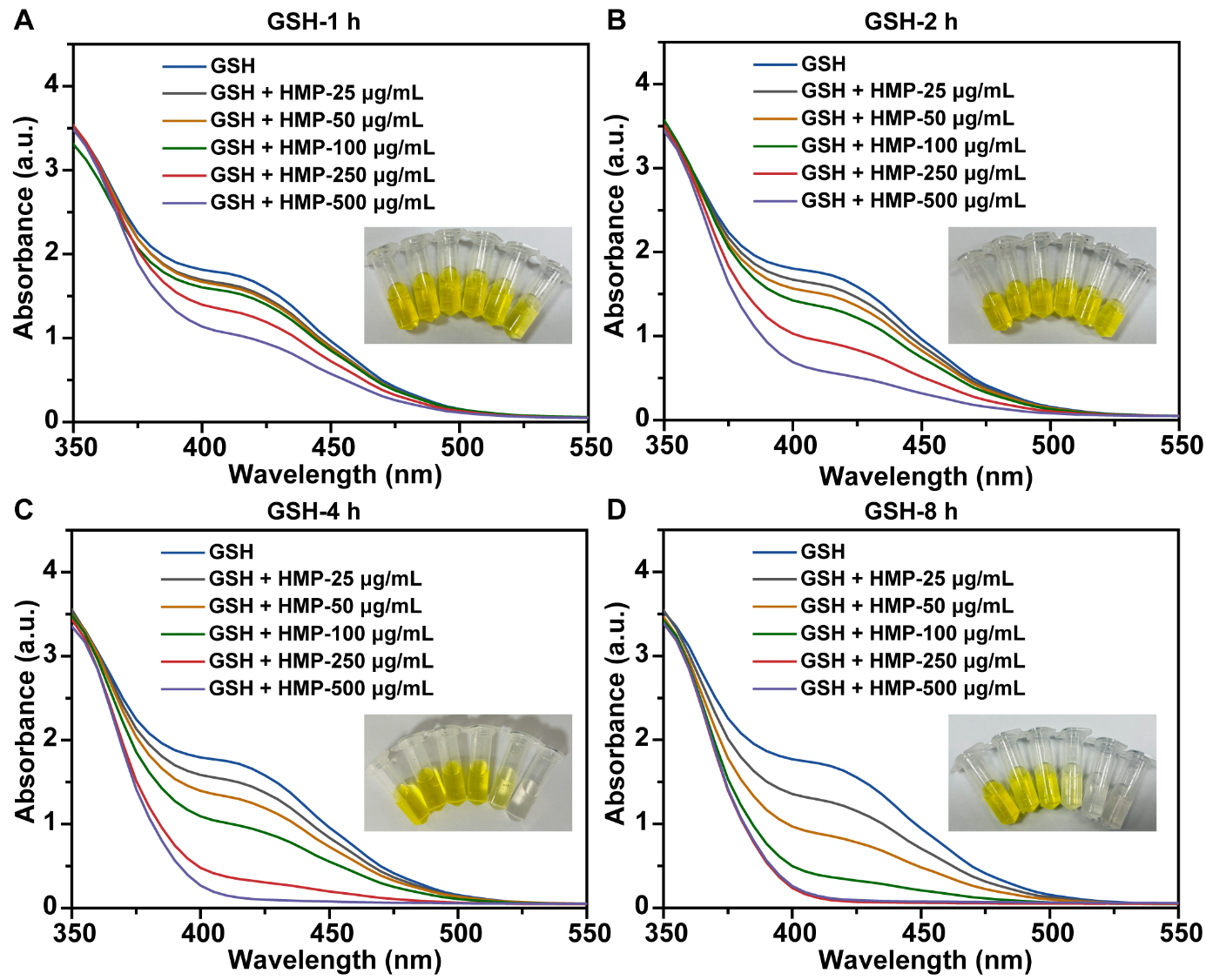


**Fig. S9** The UV-Vis absorbance and corresponding photos (inset) of time-dependent GSH consumption through redox reaction of different HMP concentrations.


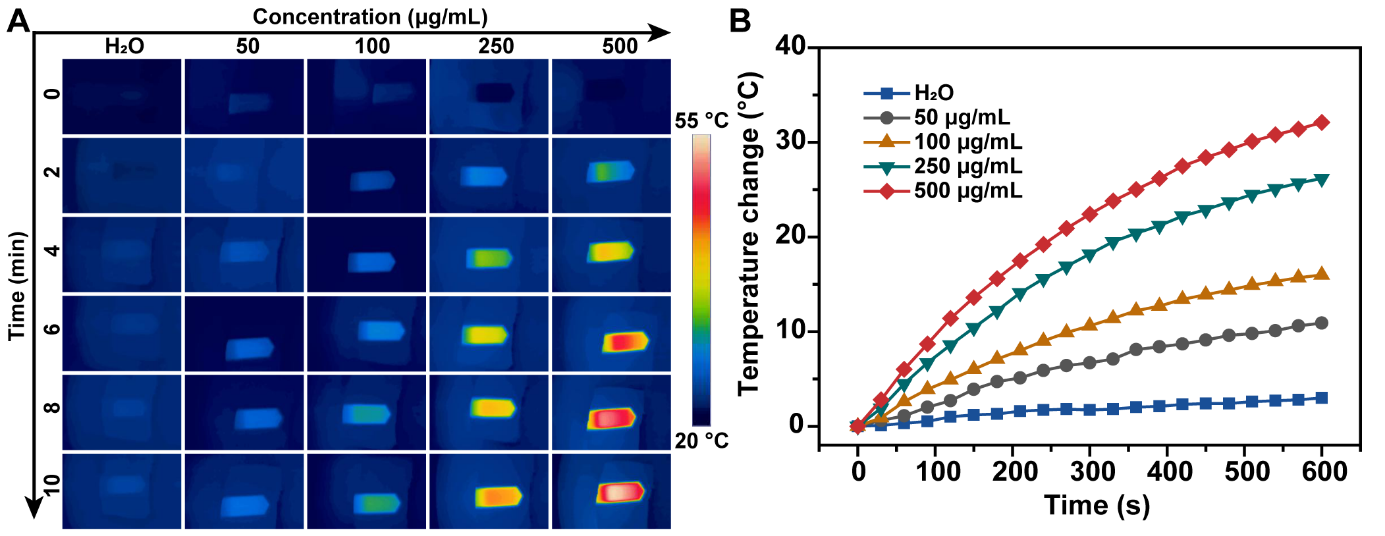


**Fig. S10** (A) Photothermal images and (B) corresponding temperature change curve of HMP with various concentration under 808-nm laser irradiation (1 W/cm^2^).


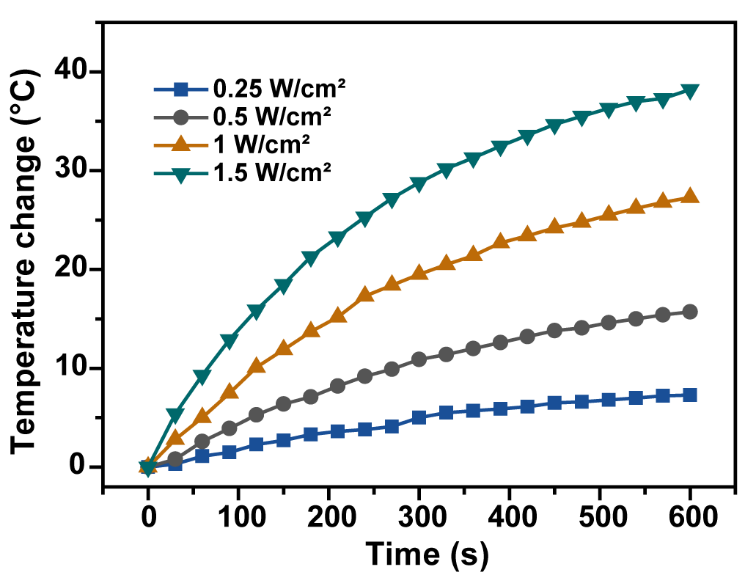


**Fig. S11** Temperature change curve of HMP (250 μg/mL) at different laser power.


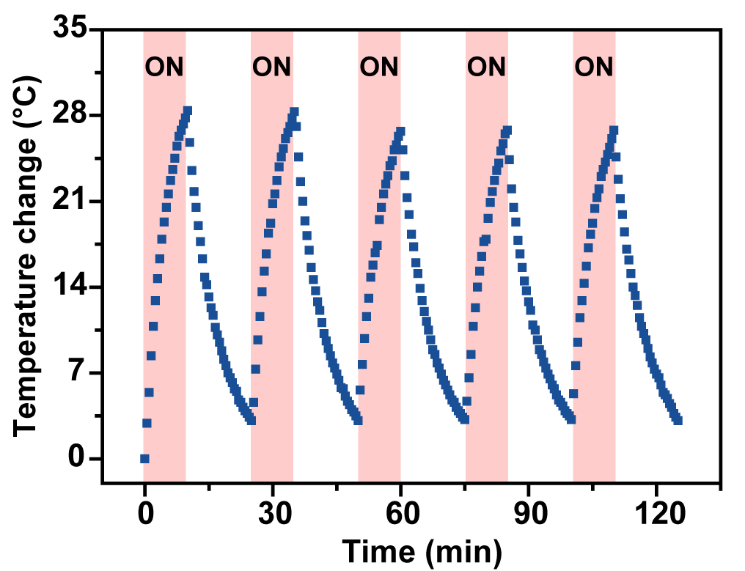


**Fig. S12** Photothermal stability of HMP (250 μg/mL) undergoing five on/off cycles of 808-nm laser irradiation (1 W/cm^2^).


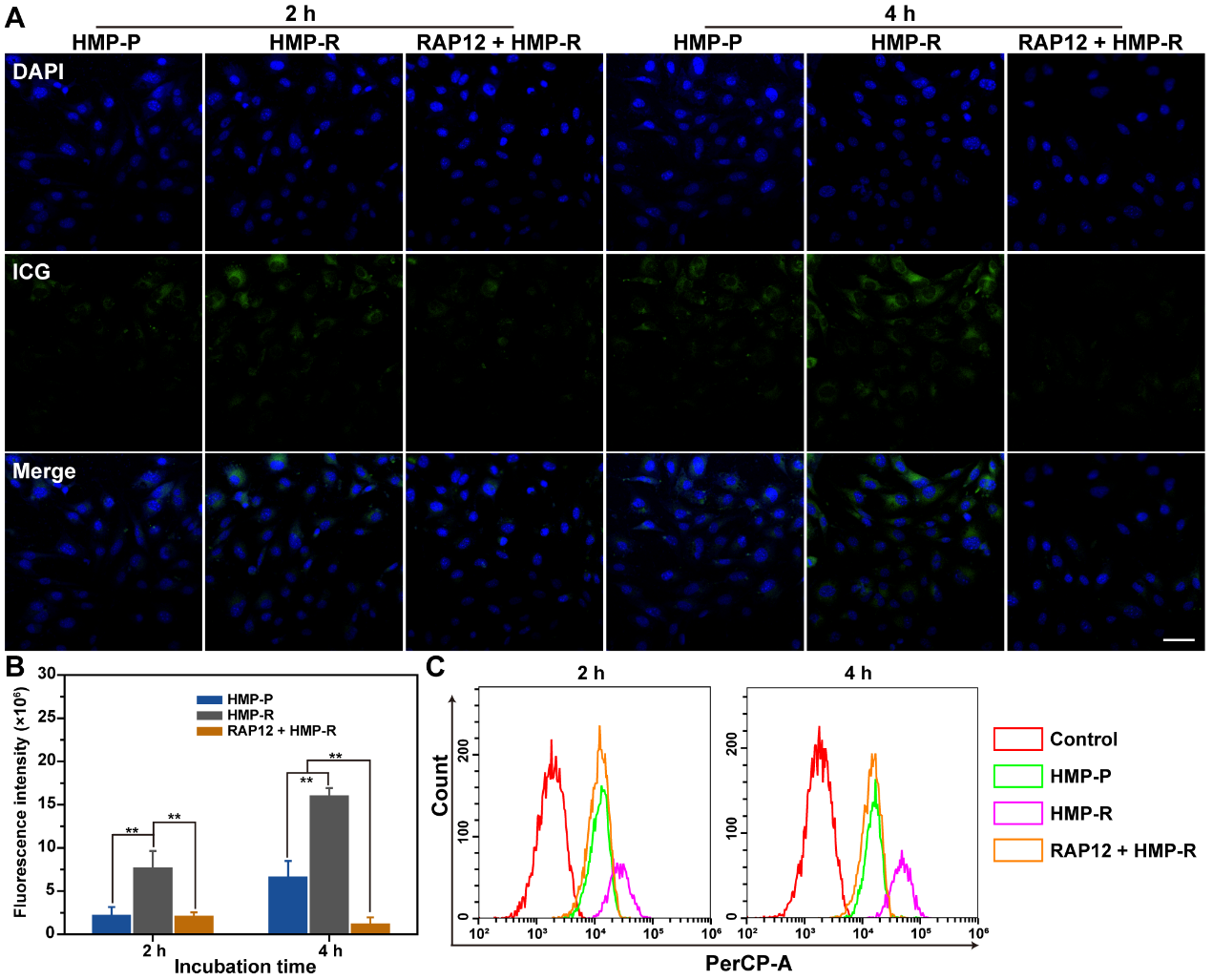
**Fig. S13** (A) Fluorescence images after bEnd.3 cells were incubated with HMP-P and HMP-R for 2 and 4 h, respectively, or RAP12 peptide (200 μg/mL) pre-treatment for 0.5 h. Scale bar: 50 μm. (B) Corresponding quantitative results of the intracellular fluorescence intensity in bEnd.3 cells. The data are presented as mean ± SD (n = 3). ^**^*P* < 0.01. (C) Flow cytometry analysis of bEnd.3 cells after 2 or 4 h of incubation. The statistical significance was calculated via one-way ANOVA Tukey’s multiple comparisons test.


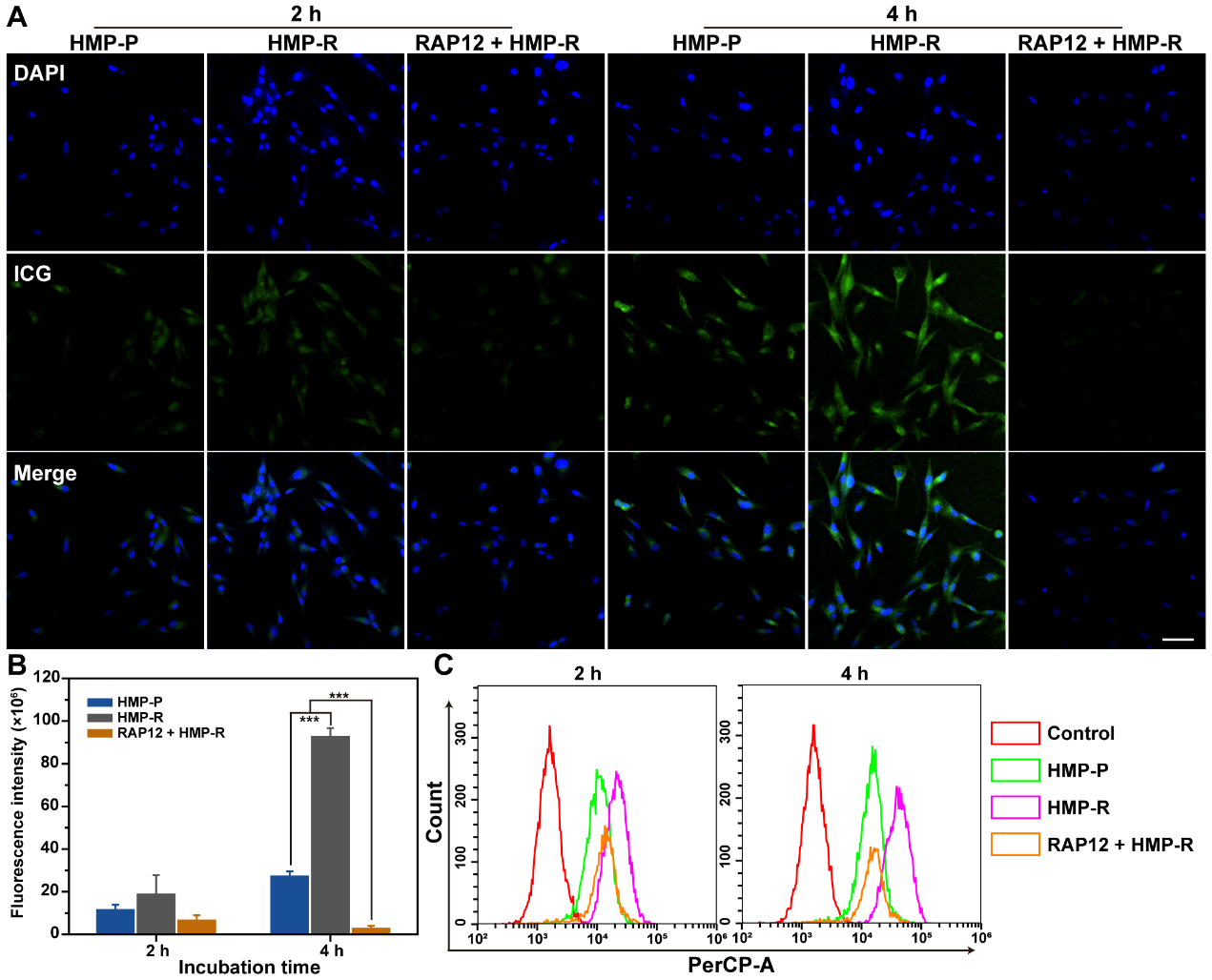


**Fig. S14** (A) Fluorescence images after U87 cells were incubated with HMP-P and HMP-R for 2 and 4 h, respectively, or RAP12 peptide (200 μg/mL) pre-treatment for 0.5 h. Scale bar: 50 μm. (B) Corresponding quantitative results of the intracellular fluorescence intensity in U87 cells. The data are presented as mean ± SD (n = 3). ^***^*P* < 0.001. (C) Flow cytometry analysis of U87 cells after 2 or 4 h of incubation. The statistical significance was calculated via one-way ANOVA Tukey’s multiple comparisons test.


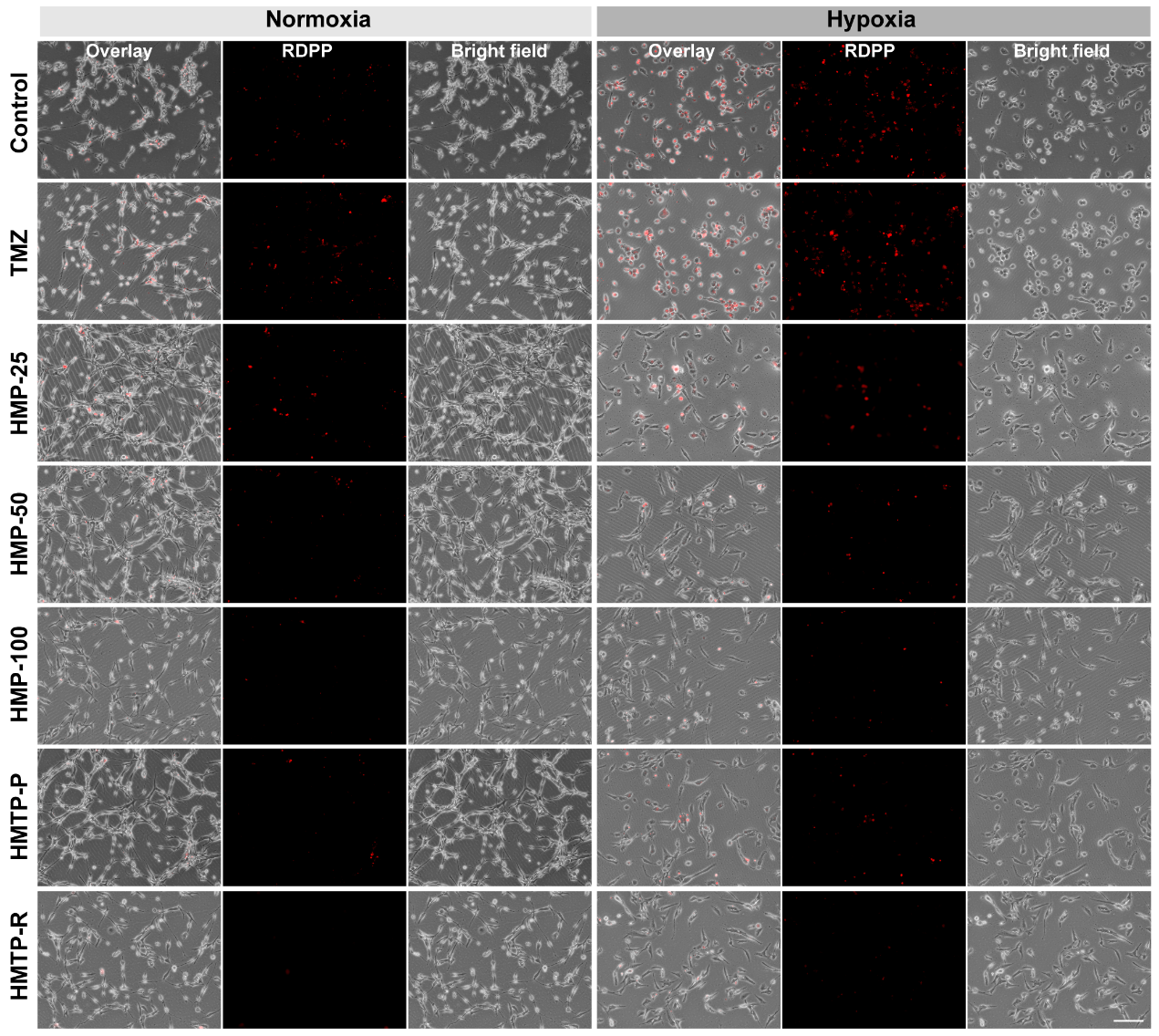


**Fig. S15** Intracellular O_2_ generation was monitored via O_2_ sensor ([Ru(dpp)_3_]Cl_2_) of U87 cells after fresh medium, TMZ, HMTP-P, HMTP-R and HMP with various concentration. Scale bar: 100 μm.


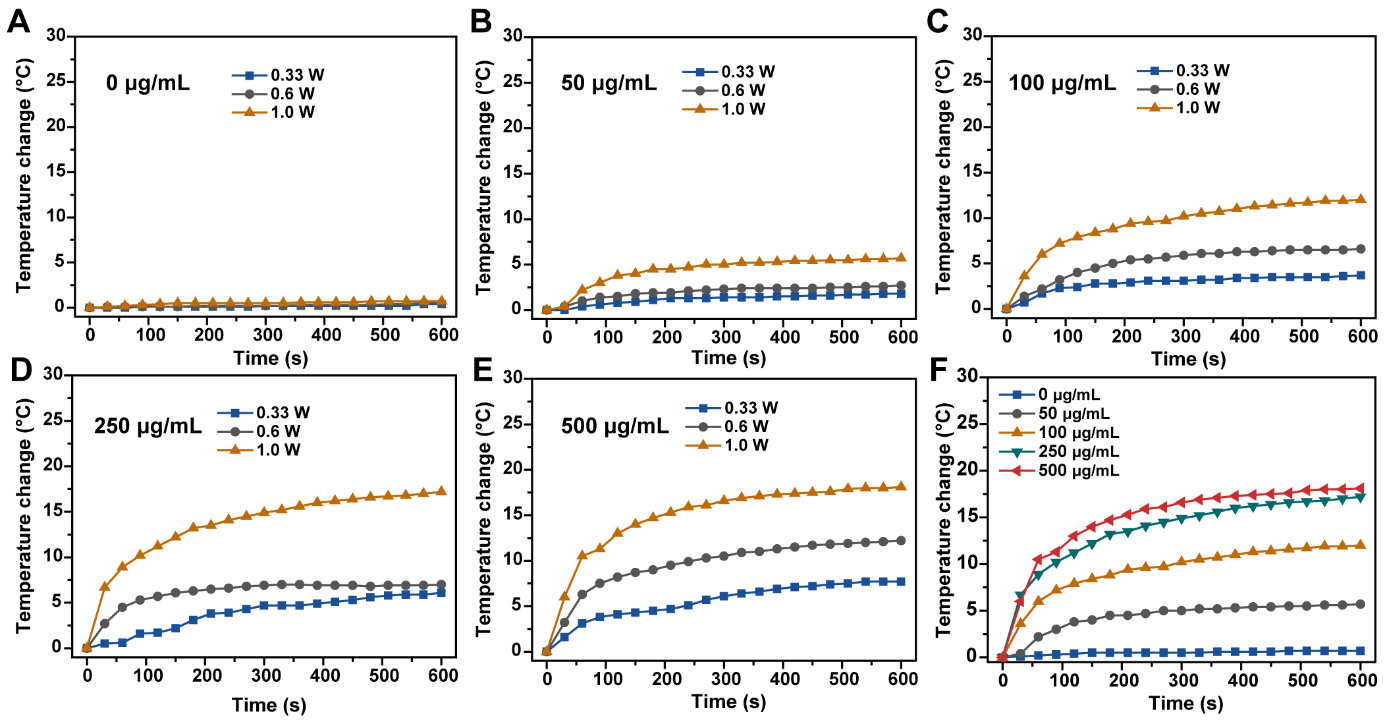


**Fig. S16** Intracellular photothermal evaluation after bEnd.3 cells were treated with various concentration of HMP and power of 808-nm laser irradiation.


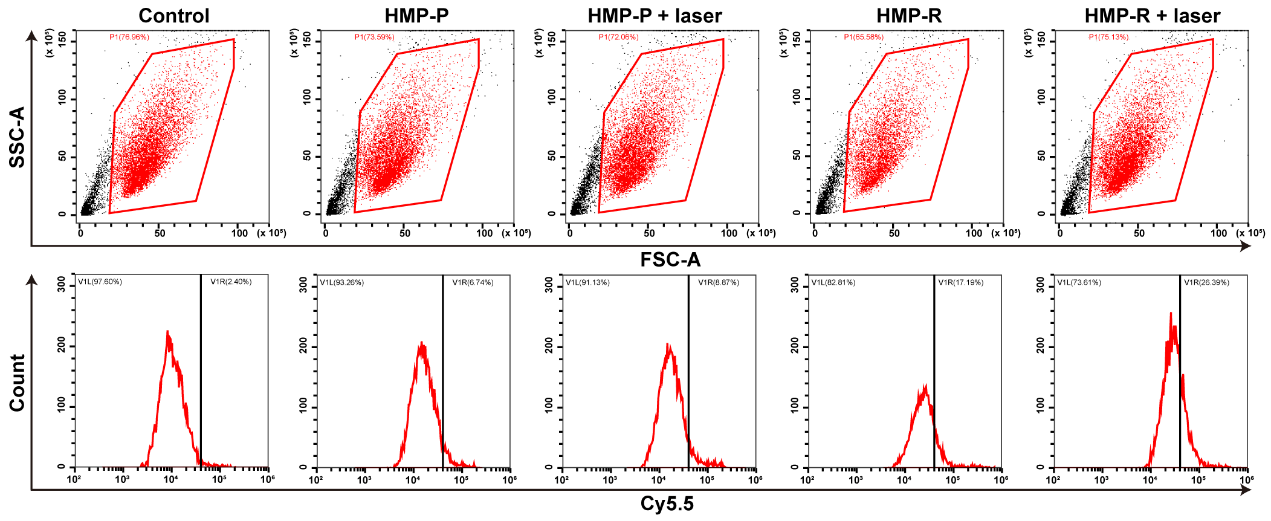


**Fig. S17** Gating strategy of flow cytometry.


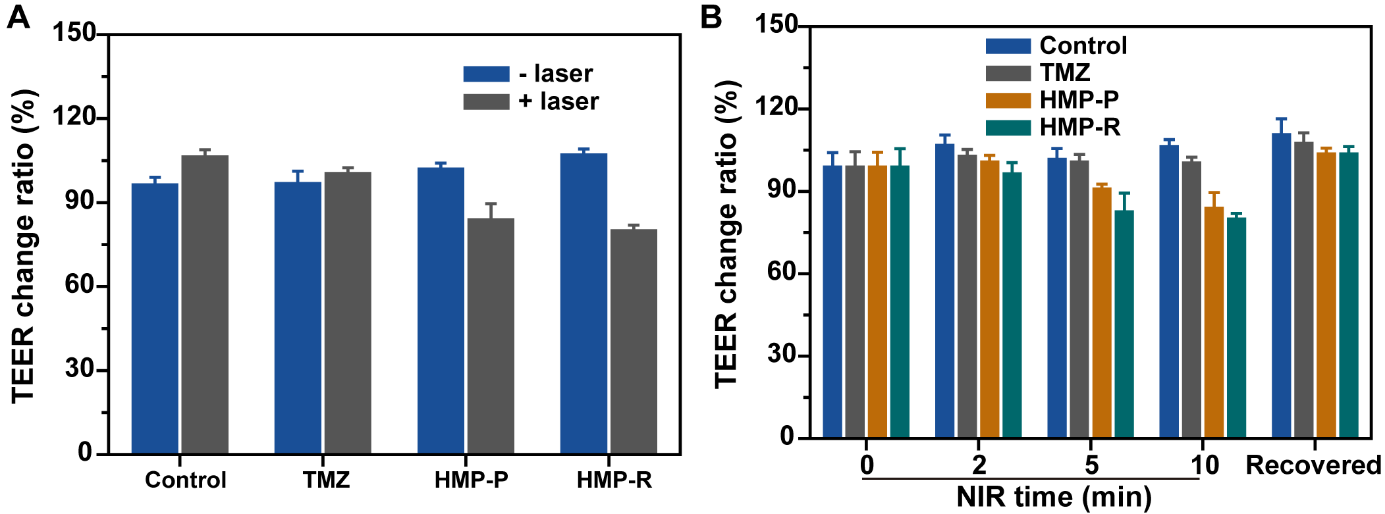


**Fig. S18** (A) TEER change ratio after fresh medium, TMZ, HMP-P, and HMP-R treatment with or without laser irradiation (808 nm, 1W/cm^2^, 10 min). (B) NIR time-dependent TEER change ratio after fresh medium, TMZ, HMP-P, and HMP-R with laser irradiation (808 nm, 1W/cm^2^, 10 min). The data are presented as mean ± SD (n = 3).


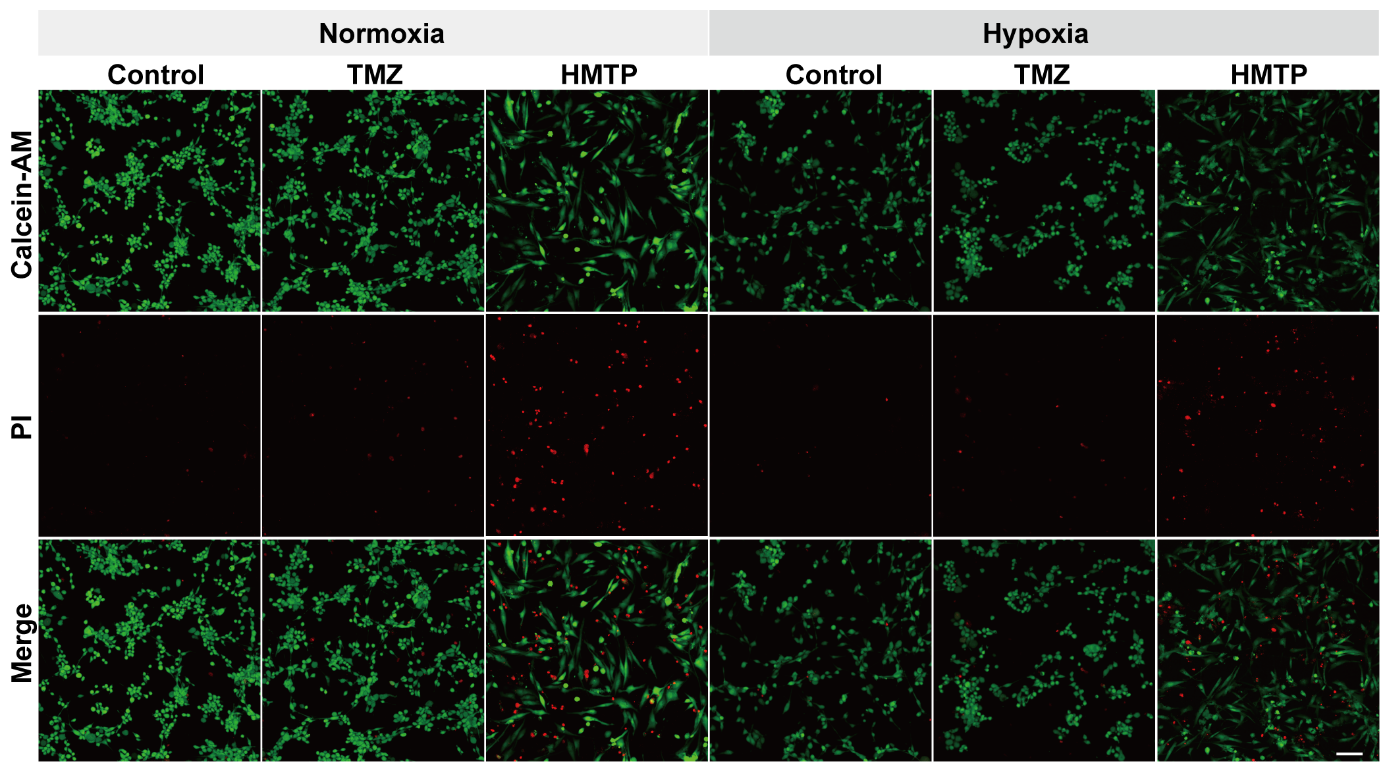


**Fig. S19** CLSM images of U87 cells co-stained with Calcein-AM (green, live cells staining) and propidium iodide (red, dead cells staining) after different treatment. Scale bar: 100 μm.


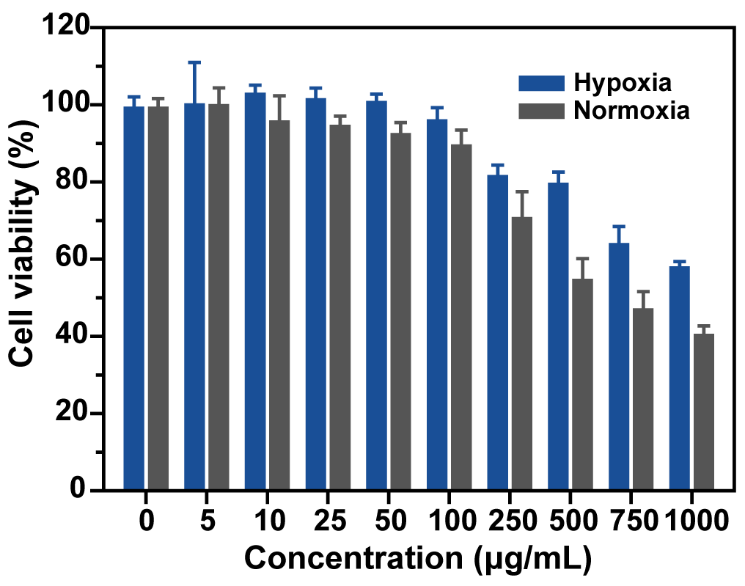


**Fig. S20** Cell viability of U87 cells after treatment with TMZ at various concentrations under hypoxia or normoxia environment. The data are presented as mean ± SD (n = 5).


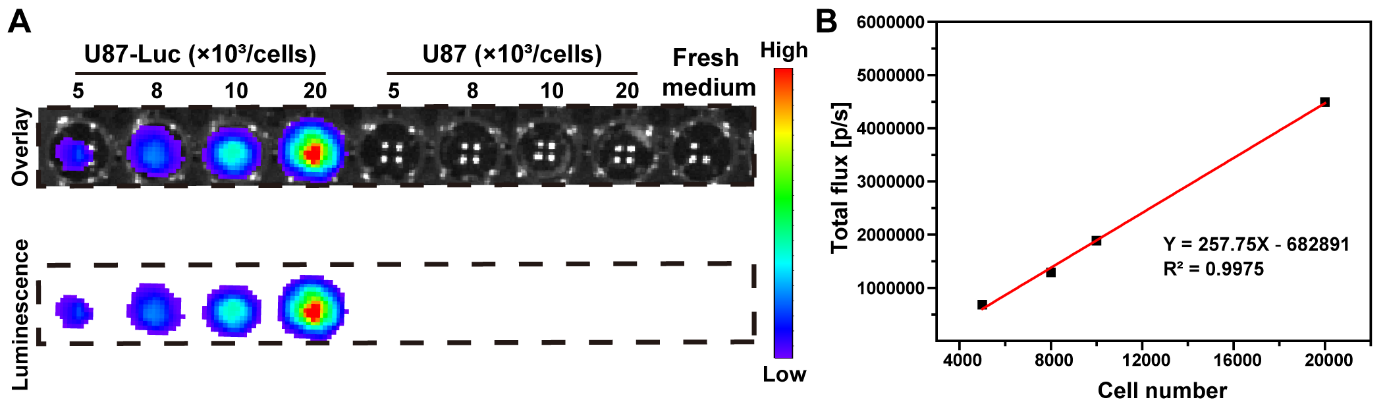


**Fig. S21** (A) Luminescence imaging of U87-Luc and U87 cells. (B) Standard curve of the total luminescence of U87-Luc cells.


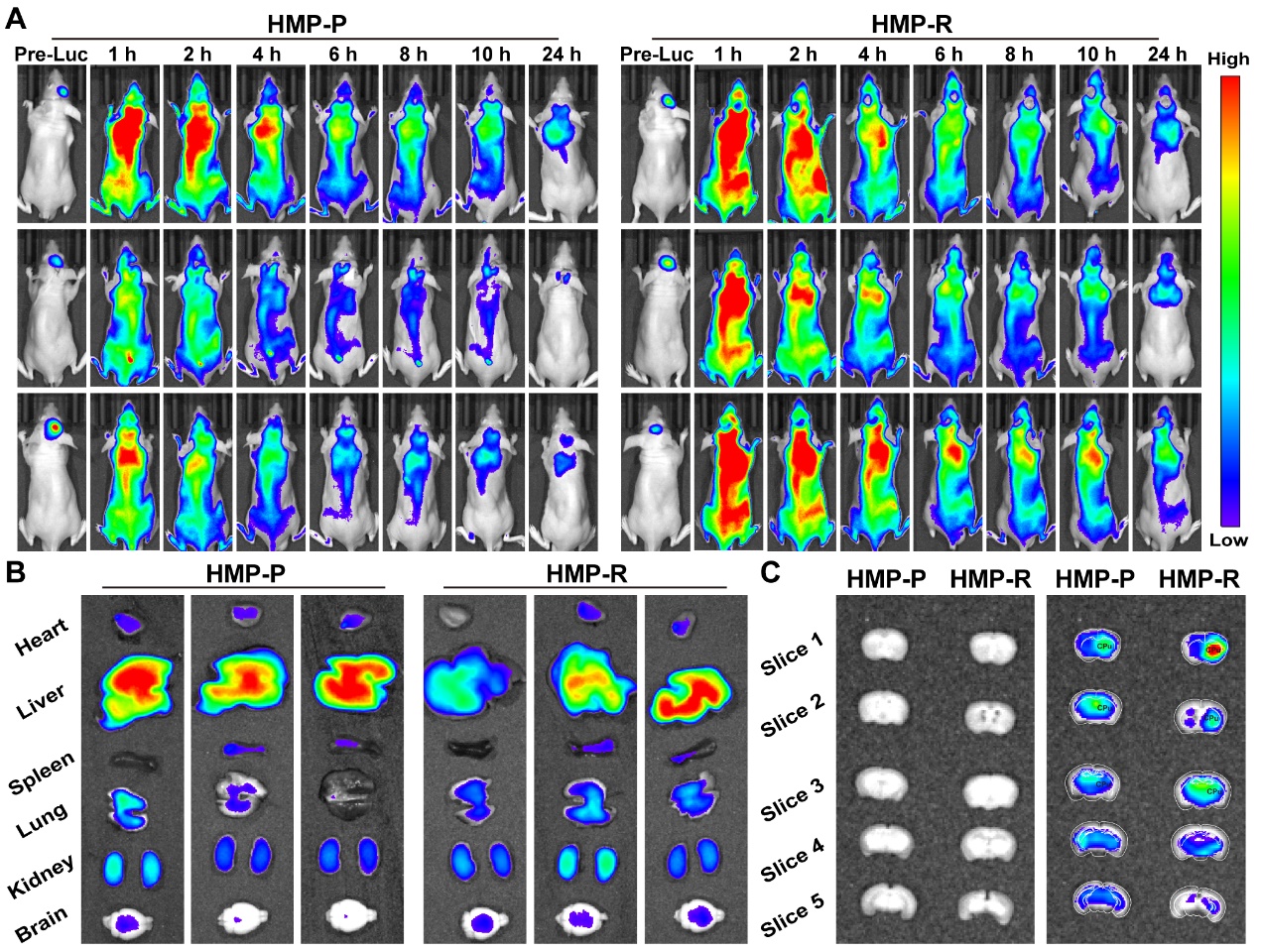


**Fig. S22** (A) Time-dependent *in vivo* fluorescence imaging of U87-tumor-bearing mice after injection of HMP-P and HMP-R (n = 3). (B) *Ex vivo* fluorescence images of major organs collected at 24 h post-injection (n = 3). (C) Overlaid image of magnified fluorescent images and Allen Brain Atlas. CPu = caudate putamen (striatum).


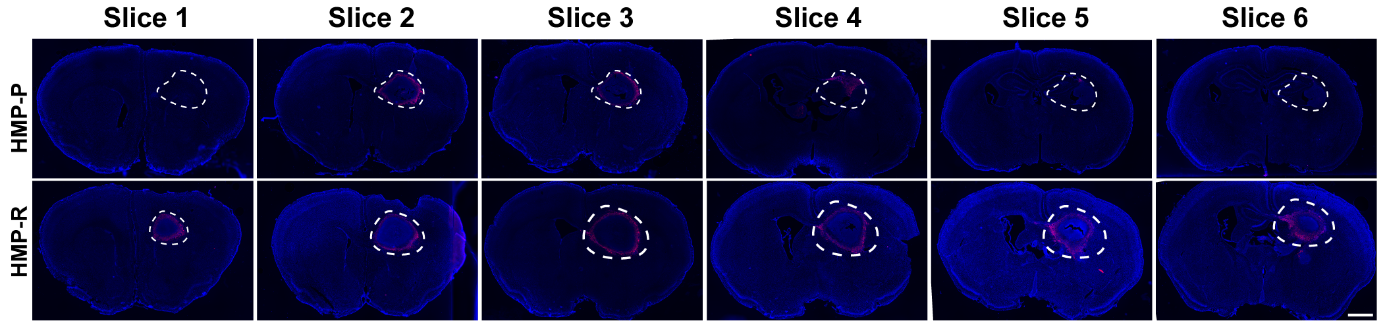


**Fig. S23** The distribution of Cy5.5-labeled nanoparticles in the brain tissues post-injection of HMP-P and HMP-R. Scale bar: 1 mm.


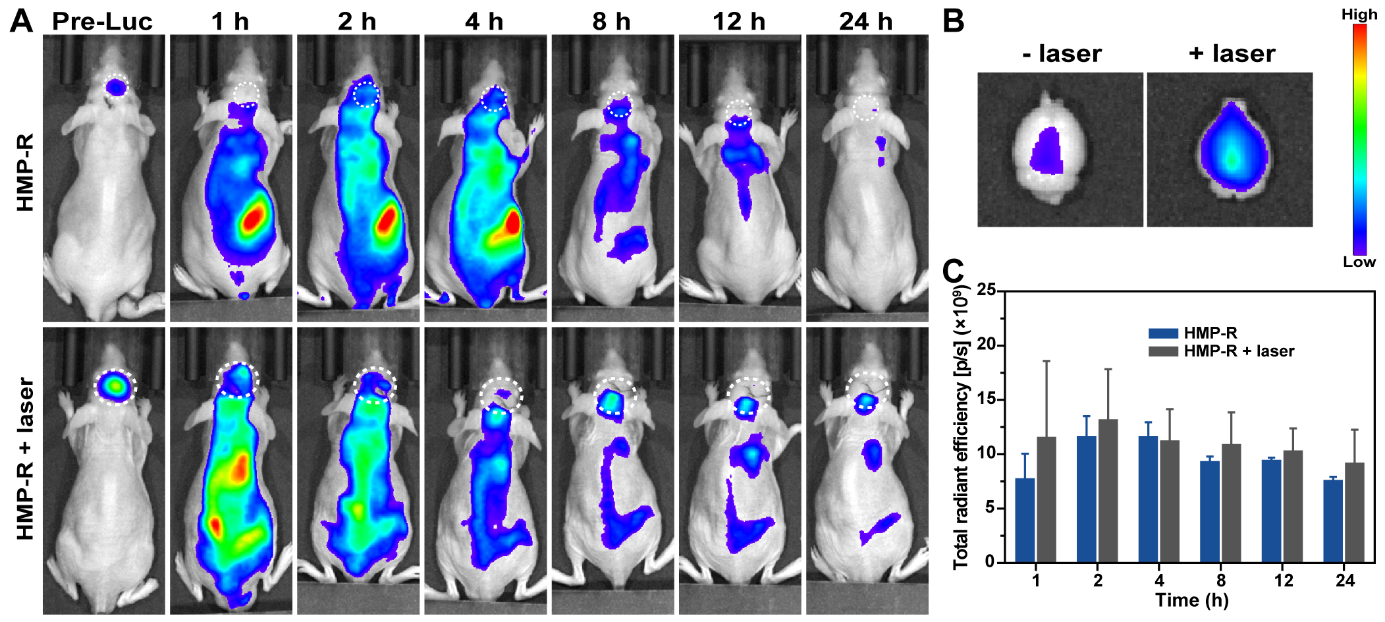


**Fig. S24** (A) Time-dependent in vivo fluorescence imaging of U87-tumor-bearing mice after intravenous injection of HMP-R with or without laser irradiation at different time points. (B) *Ex vivo* fluorescence images of brain collected at 24 h post-injection. (C) Relative fluorescence intensity of the *ex vivo* brains. The data are presented as mean ± SD (n = 3).


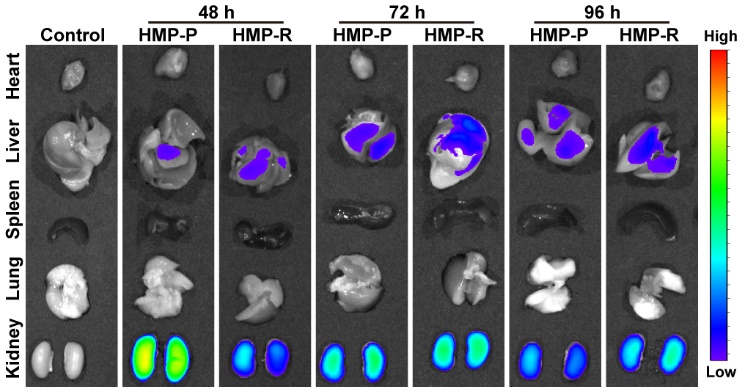


**Fig. S25** *Ex vivo* imaging of the major organs of mice treated with HMP-P or HMP-R for 48, 72 or 96 h.


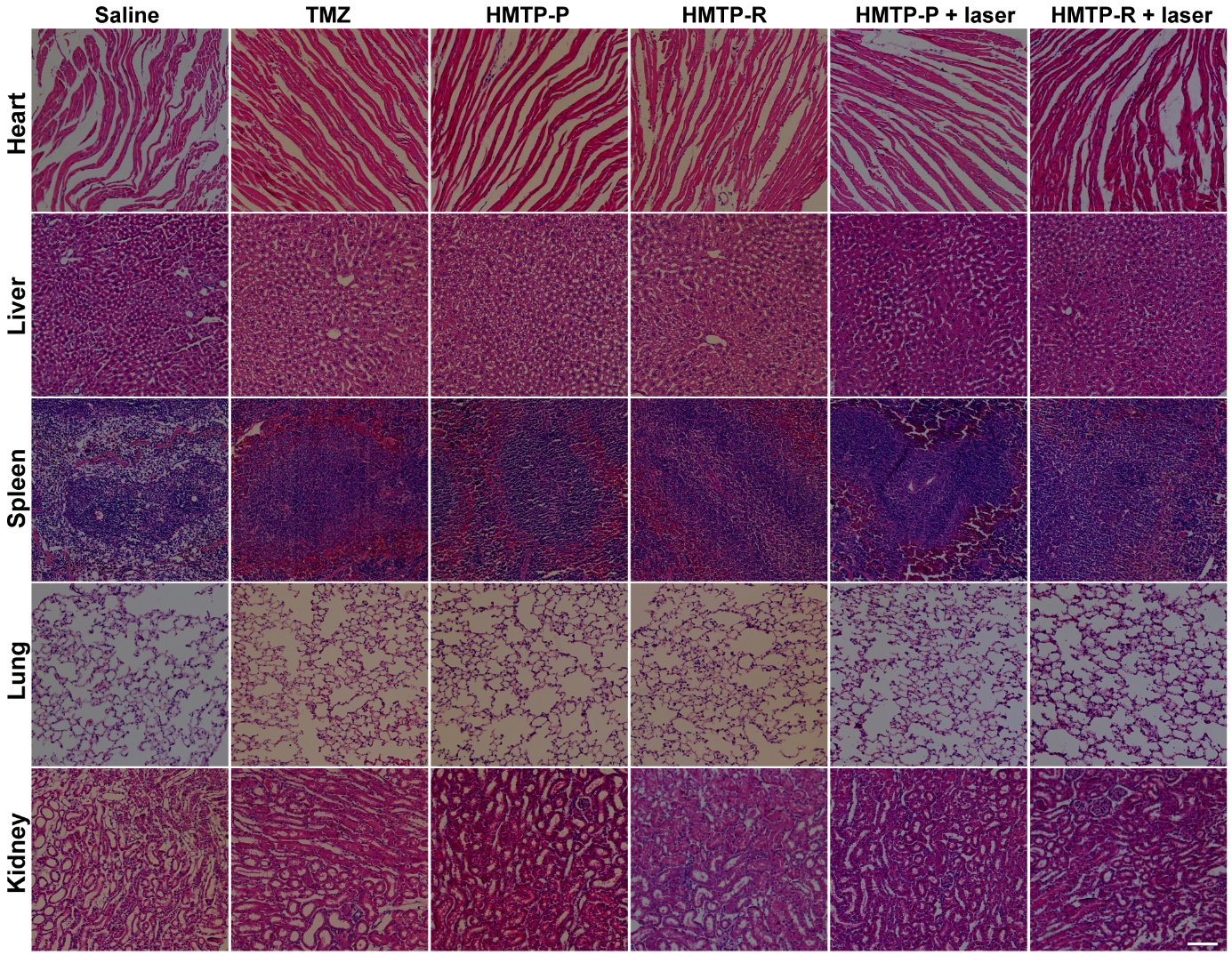


**Fig. S26** H&E staining of major organs after different treatments. Scale bar: 100 μm.


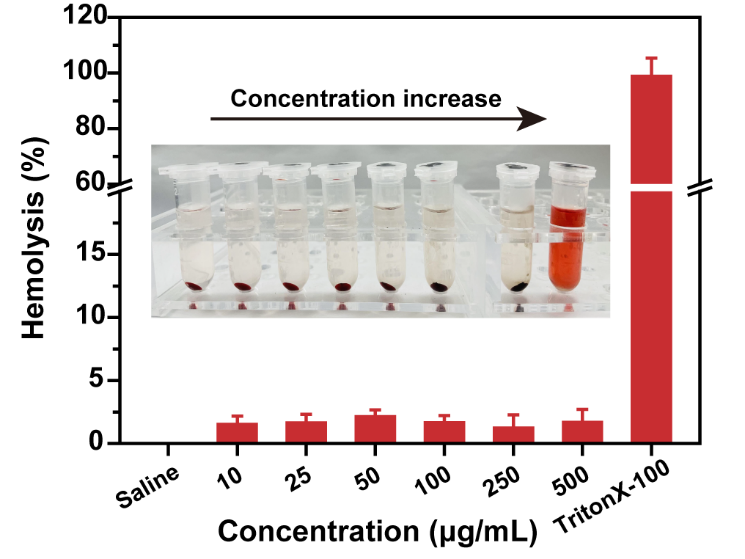


**Fig. S27** Hemolysis analysis of HMP at various concentrations. RBCs in PBS and TritonX-100 were set as the negative control and positive control, respectively (n = 3).


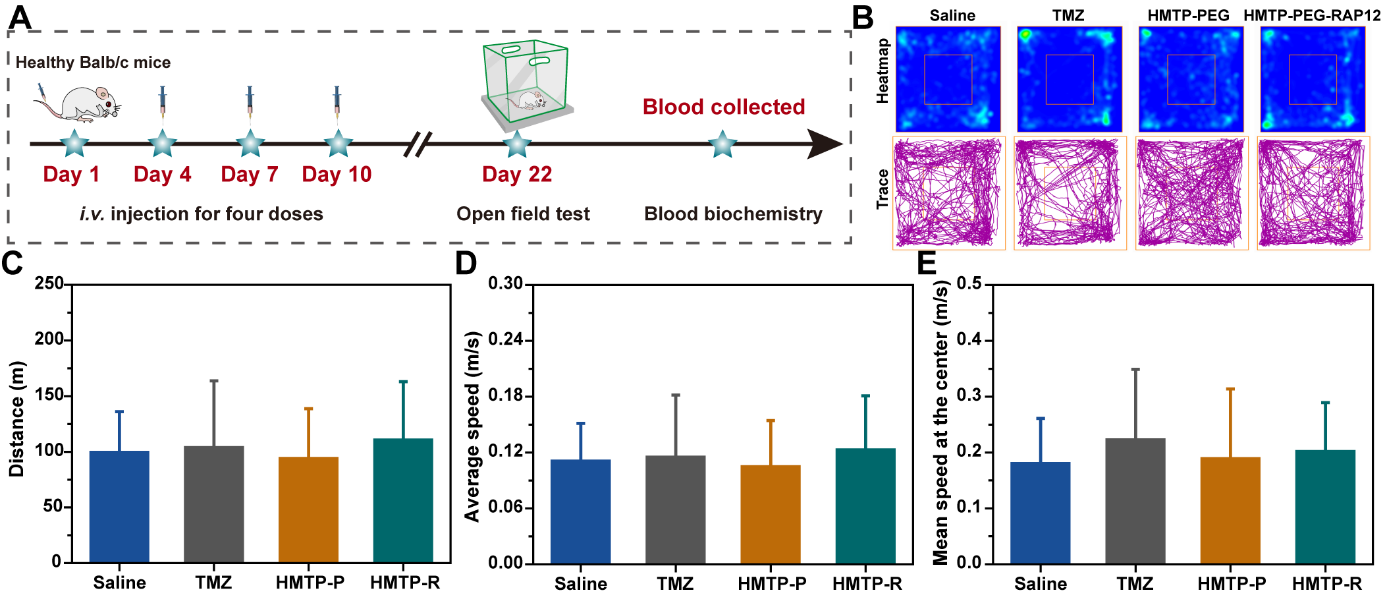


**Fig. S28** (A) Schematic illustration of experimental scheme of open-field test. (B) Heatmap and typical motion route during an open-field test after saline, TMZ, HMTP-P, and HMTP-R treatment. (C) The distance, (D) average speed, and (E) mean speed at the center in 15 min for mice in each group. The data are presented as mean ± SD (n = 6).


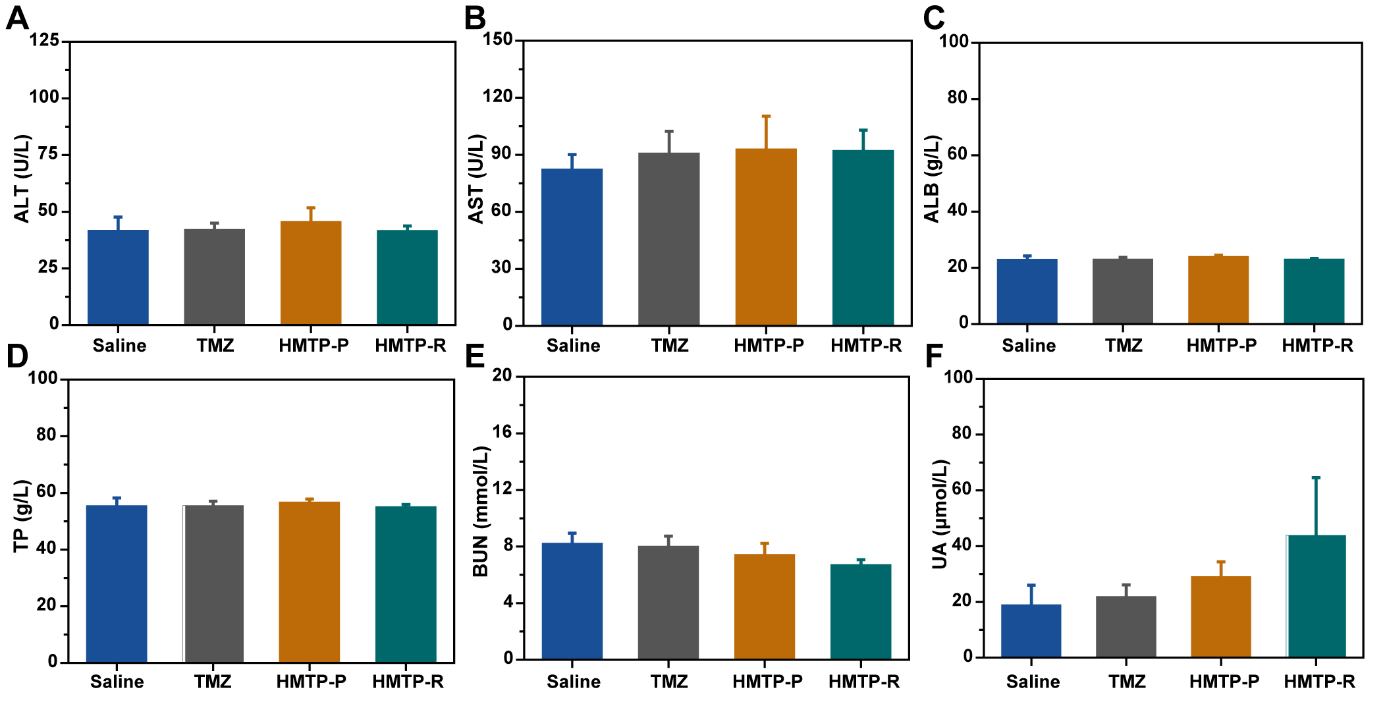


**Fig. S29** (A) Plasma alanine aminotransferase (ALT), (B) aspartate aminotransferase (AST), (C) albumin (ALB), (D) total protein (TP), (E) blood urea nitrogen (BUN), and (F) uric acid (UA) in healthy Balb/c mice treated with saline, TMZ, HMTP-P, and HMTP-R. The data are presented as mean ± SD (n = 6).
